# Normality sensing licenses local T cells for innate-like tissue surveillance

**DOI:** 10.1038/s41590-021-01124-8

**Published:** 2022-02-14

**Authors:** Duncan R. McKenzie, Rosie Hart, Nourdine Bah, Dmitry S. Ushakov, Miguel Muñoz-Ruiz, Regina Feederle, Adrian C. Hayday

**Affiliations:** 1 The Francis Crick Institute, London, UK; 2 Peter Gorer Department of Immunobiology, King’s College London, London, UK; 3 Monoclonal Antibody Core Facility, Helmholtz Zentrum München, Neuherberg, Germany; 4 Present Address: Institute of Molecular Virology and Cell Biology, Friedrich-Loeffler-Institut, Federal Research Institute for Animal Health, Greifswald-Insel Riems, Germany

**Keywords:** Immunological surveillance, Gammadelta T cells, Lymphocyte activation

## Abstract

The increasing implication of lymphocytes in general physiology and immune surveillance outside of infection poses the question of how their antigen receptors might be involved. Here, we show that macromolecular aggregates of intraepidermal γδ T cell antigen receptors (TCRs) in the mouse skin aligned with and depended on Skint1, a butyrophilin-like (BTNL) protein expressed by differentiated keratinocytes (KCs) at steady state. Interruption of TCR-mediated ‘normality sensing’ had no impact on γδ T cell numbers but altered their signature phenotype, while the epidermal barrier function was compromised. In addition to the regulation of steady-state physiology, normality sensing licensed intraepidermal T cells to respond rapidly to subsequent tissue perturbation by using innate tumor necrosis factor (TNF) superfamily receptors. Thus, interfering with Skint1-dependent interactions between local γδ T cells and KCs at steady state increased the susceptibility to ultraviolet B radiation (UVR)-induced DNA damage and inflammation, two cancer-disposing factors.

## Abstract (web summary)

Hayday and colleagues show that sustained Skint1-dependent interactions between murine intraepidermal γδ T cells and keratinocytes are required to maintain the homeostatic barrier function and phenotype of the intraepidermal γδ T cells, including their preparedness to respond appropriately to epidermal challenges.

## Main

The steady-state immune system was long thought to be resting, awakened only by infection. However, this perspective has been transformed by examples of immune responses to non-microbial challenges and evidence that the steady-state immune system helps maintain physiological processes from metabolism to memory and barrier integrity^1–4^. Many such interactions happen locally, involving immune cells permanently associated with non-lymphoid tissues^5–7^. While macrophages are well-established regulators of tissue status, an increasing implication of lymphocytes in this process raises the question of whether antigen receptors are used to sense tissue perturbation, akin to recognition of foreign antigens.

To address this, we have focused on intraepithelial lymphocytes (IELs), which compose one of the largest T cell compartments and are variably conserved in multiple tissues from agnathans to humans^8,9^. IELs are commonly implicated in tissue stress surveillance but with little mechanistic understanding^10–12^. IELs are disproportionately enriched in γδ T cells, which express site-specific TCRs, for example, V_γ_5V_δ_1 in mouse epidermis, V_γ_7V_δ_*n* in mouse intestine and V_γ_4V_δ_1 in human intestine^9,13–15^. Such selective repertoire focusing reflects the developmental dependence of IELs on cognate tissue-specific, epithelial-expressed BTNL heteromers. Thus, Skint1 and Skint2, two PD-L1-like members of the BTNL family, select murine V_γ_5V_δ_1^+^ IELs, Btnl1 and Btnl6 select murine V_γ_7^+^ intestinal IELs, and BTNL3 and BTNL8 selectively regulate human colonic V_γ_4^+^ IELs^15–19^. Indeed, BTNL3 is a bona fide TCRγδ ligand^20,21^.

Whereas Skint1 and Skint2 mediate V_γ_5V_δ_1^+^ precursor development in the thymus, they are also expressed by suprabasal KCs, among which mature V_γ_5V_δ_1^+^ IELs permanently reside^17^. How they might continue to influence mature IEL function is unknown. In that regard, focal clusters of V_γ_5V_δ_1^+^ TCRs constitutively engage KCs at steady state and may engage in signaling^22^, but neither the epithelial determinant(s) nor the biological implications of these engagements is understood.

Here, we focused on V_γ_5V_δ_1^+^ IELs, also termed dendritic epidermal T cells (DETCs), to investigate how tissue T cells might use TCRs to distinguish normal self from stressed self. We show that constitutive DETC TCR-containing foci^22^ align with and depend upon sustained epithelial expression of Skint1. Although these interactions did not maintain DETC numbers, they preserved DETC and KC gene expression patterns and maintained steady-state epithelial barrier function. Additionally, these interactions licensed DETCs to utilize costimulatory receptors to make rapid responses to UVR and chemical irritation. Thus, interruption of steady-state, TCR-mediated DETC–KC interactions increased epidermal susceptibility to sustained DNA damage and inflammation, two factors that jointly dispose to cancer^23^. As Skint1-dependent DETC–KC interactions occur at steady state, this mode of tissue immunosurveillance can be viewed as ‘normality sensing’. This offers a revised perspective of tissue immunosurveillance and of antigen receptor biology, which conventionally focuses on the recognition of molecules induced by change.

## Results

### DETCs sense steady-state KCs via Skint1

**Fig. 1:**
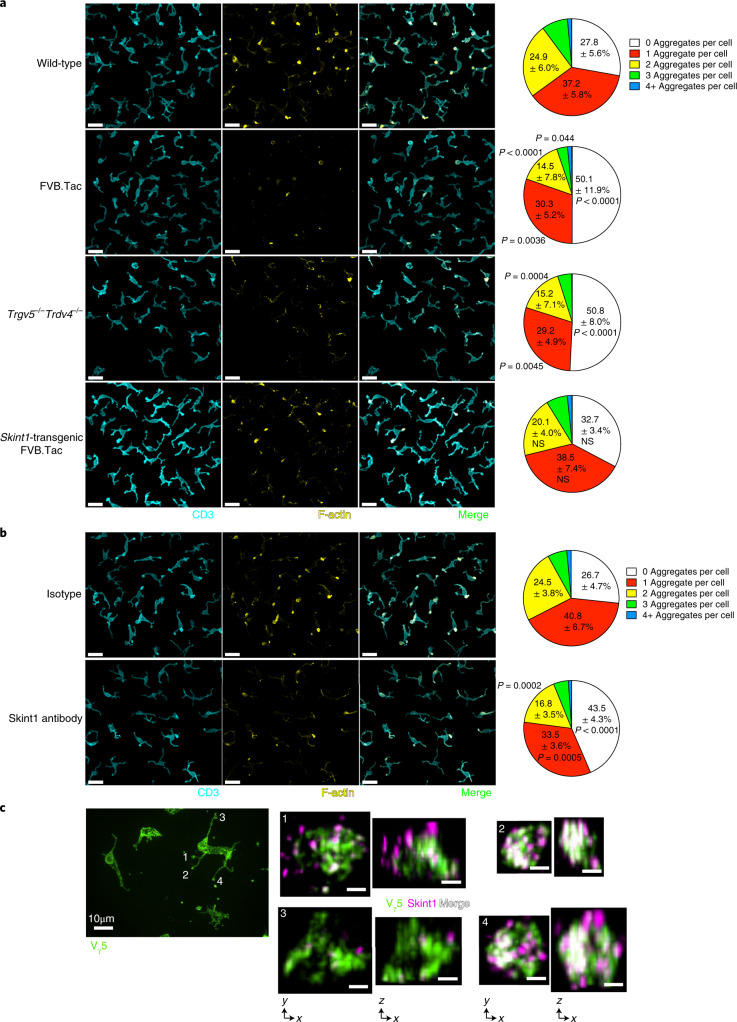
Steady-state DETC–KC interactions depend on the V_γ_5V_δ_1 TCR and Skint1. **a**,**b**, Confocal microscopy of CD3^+^ DETCs (cyan) and F-actin within a CD3 mask (yellow) in epidermal sheets. The frequency distribution of the number of interacting dendrites (CD3- and F-actin-rich aggregates) per cell is shown, using mean values per mouse. Interacting dendrites from wild-type FVB (*n* = 16), FVB.Tac (*n* = 12), *Trgv5*^–/–^*Trdv4*^–/–^ (*n* = 7) and *Skint1*-transgenic FVB.Tac mice (*n* = 12) are shown in **a**; scale bar, 20 μm. Interacting dendrites from wild-type FVB mice administered Skint1 antibody (*n* = 10) or isotype control (*n* = 9) by intradermal (i.d.) injection 6 h before are shown in **b**; scale bar, 20 μm. **c**, Instant structured illumination microscopy (iSIM) of whole-mount epidermal sheets from unchallenged wild-type FVB mice. Left, V_γ_5 TCR (green) staining; scale bar, 10 μm. Right, top–down and lateral views of three-dimensional (3D) reconstructions of typical V_γ_5 TCR (green) and Skint1 (magenta) staining in selected dendrite tips from the left-hand image; scale bar, 1 μm. Representative images and pooled data are from eight (**a**) or three (**b** and **c**) independent experiments. A matched two-way analysis of variance (ANOVA) with Dunnett’s multiple comparisons test relative to wild-type (**a**) or a Sidak’s multiple comparisons test (**b**) was used to analyze the data. Values in pie charts represent mean ± s.d.; NS, not significant. Source data

**Extended Data Fig. 1:**
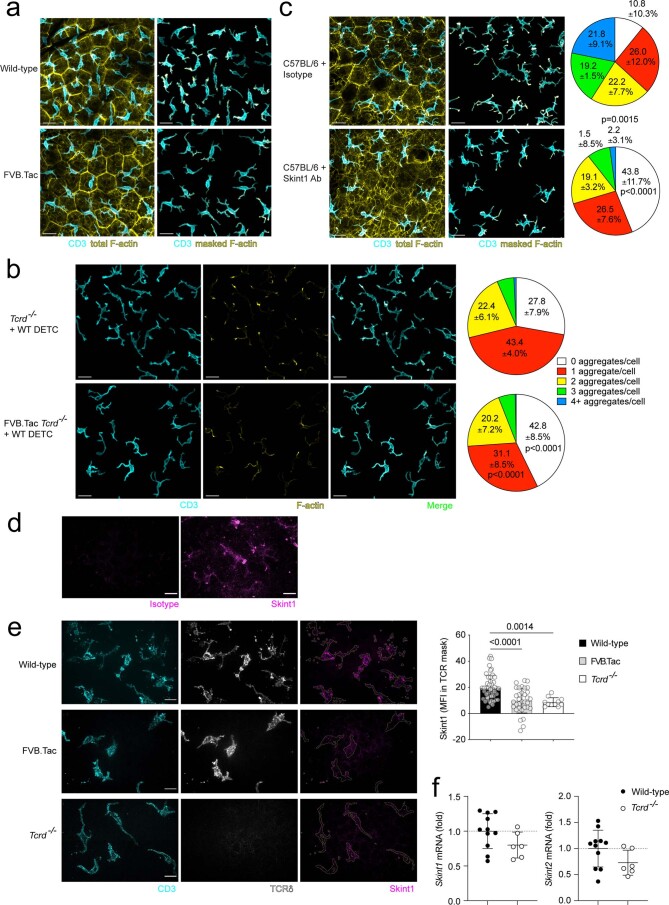
Steady state DETC-keratinocyte interactions depend on the Vγ5Vδ1 TCR and Skint1. **a)** Confocal microscopy of CD3^+^ DETCs (cyan) and total F-actin or that within CD3 mask (yellow) in epidermal sheets from wild-type and FVB.Tac mice. Representative of n=4/group. Scale bar 20μm. **b**) Neonatal *Tcrd*^*-/-*^ (n=13) and FVB.Tac.*Tcrd*^*-/-*^ (n=11) pups were reconstituted with wild-type embryonic thymocytes containing mature Skint1-selected DETC precursors. Confocal microscopy of CD3^+^ DETCs (cyan) and F-actin within CD3 mask (yellow) in epidermal sheets at 8 weeks of age. Scale bar 20μm. Frequency distribution of number of interacting dendrites within Vγ5^+^ DETCs (CD3- and F-actin-rich aggregates) per cell using mean values per mouse. **c)** Confocal microscopy of CD3^+^ DETCs (cyan) and total F-actin or that within CD3 mask (yellow) in epidermal sheets from wild-type C57BL/6 mice pre-treated with Skint1 Ab or isotype control (n=5/group). Scale bar 20μm. Frequency distribution of number of interacting dendrites (CD3- and F-actin-rich aggregates) per cell using mean values per mouse. **d)** Instant structured illumination microscopy (iSIM) of Skint1 (magenta) or isotype control in whole mount epidermal sheets from wild-type FVB mice. Scale bar 10μm. **e**) iSIM of CD3 (cyan), TCRδ (white) and Skint1 (magenta) staining in whole mount epidermal sheets from wild-type FVB(n=3), FVB.Tac (n=5) and *Tcrd*^*-/-*^ (n=2) mice. Scale bar 10μm. Mean Skint1 MFI quantified within TCRδ (wild-type, FVB.Tac) or CD3 (*Tcrd*^*-/-*^) masks relative to mean image background value. Each dot represents a single cell. **f)** qPCR for *Skint* gene expression from epidermis of wild-type FVB (n=11) and *Tcrd*^*-/-*^ mice (n=6). Representative images of and cumulative data from 4 (**b**) or 3 (**e**) independent experiments. Pooled data from 2 independent experiments (**f**). Matched two-way (**b-c**) or ordinary one-way (**e**) ANOVA with Sidak’s multiple comparisons test, unpaired two-tailed t-test (**f**). Values in pie charts and error bars on graph represent mean ± SD. ns: not significant. Source data

At steady state, V_γ_5V_δ_1^+^ TCRs focus at apical dendrite tips in supramolecular clusters that contact neighboring suprabasal KCs, seemingly inducing constitutive TCR signaling^22^. TCRs within these clusters colocalized with actin (Fig. 1a), as reported^22^, and most DETCs in FVB mice displayed one to four clusters (Fig. 1a). For clarity, we displayed F-actin staining masked by DETC bodies, although examination of raw staining confirmed these observations (Extended Data Fig. 1a). We next examined FVB.Tac mice, which express a truncated non-functional *Skint1*, and *Trgv5*^–/–^*Trdv4*^–/–^ mice. These two strains lack canonical V_γ_5V_δ_1^+^ DETCs^12,16^. Relative to wild-type mice, both strains showed increased frequencies of DETCs that lacked TCR–actin colocalization (Fig. 1a and Extended Data Fig. 1a). Transgenic expression of wild-type *Skint1* in FVB.Tac mice returned TCR–actin colocalization to wild-type levels (Fig. 1a). Because DETCs in FVB.Tac and *Trgv5*^–/–^*Trdv4*^–/–^ mice use non-V_γ_5V_δ_1^+^ TCRs, these results confirm reports that juxtaepithelial TCR foci require the V_γ_5V_δ_1 TCR^22^.

To identify the epithelial determinant of V_γ_5V_δ_1-dependent KC–DETC interactions, we focused on Skint1, given its critical and selective contribution to fetal thymic V_γ_5V_δ_1^+^ DETC development and its expression by differentiated suprabasal KCs^17,24^. To limit *Skint1* deficiency to the epidermis, we adoptively transferred wild-type embryonic day 16 (E16)–E18 fetal thymocytes, which include Skint1-selected CD45RB^hi^ DETC progenitors^16^, into neonatal *Tcrd*^*–/–*^ or FVB.Tac *Tcrd*^–/–^ mice. Epidermal V_γ_5^+^ DETCs formed in both hosts (Extended Data Fig. 1b), suggesting that epidermal Skint1 was not strictly necessary for the epidermal seeding of DETCs. While DETCs in *Tcrd*^*–/–*^ hosts displayed bright colocalized TCR–actin foci resembling wild-type mice, markedly fewer foci were detected in DETCs in FVB.Tac *Tcrd*^*–/–*^ mice (Extended Data Fig. 1b), strongly suggesting that Skint1 was required to establish and/or maintain juxtaepithelial TCR focusing.

To investigate whether Skint1 protein acutely maintained TCR–KC interactions, we performed an i.d. administration of 2G2, a monoclonal antibody specific for Skint1 (ref. ^19^), to wild-type FVB mice. Within 6 h after injection, the frequency of TCR–actin foci was significantly reduced compared to mice administered an isotype control antibody (Fig. 1b). A similar impact was seen in C57BL/6 wild-type mice (Extended Data Fig. 1c). We next applied iSIM to steady-state epidermal sheets from wild-type mice using Skint1 antibody staining. Despite *Skint1* expression being restricted to KCs^17^, we only detected Skint1 protein in clusters nucleated by the TCR (Fig. 1c and Extended Data Fig. 1d). Compared to wild-type mice, TCR-proximal Skint1 antibody staining was significantly reduced in FVB.Tac and *Tcrd*^*–/–*^ mice (Extended Data Fig. 1e). While truncated Skint1 in FVB.Tac mice could affect antibody staining, *Tcrd*^*–/–*^ mice expressed *Skint1* mRNA at relatively normal levels in the epidermis (Extended Data Fig. 1f), indicating that wild-type Skint1 protein was not nucleated by TCRαβ^+^ cells in the *Tcrd*^*–/–*^ epidermis^25^. Thus, Skint1 maintained steady-state TCR-mediated interactions of DETCs with KCs, which in turn shaped the epidermal localization of Skint1.

### Skint1 promotes DETC maintenance of epidermal integrity

**Fig. 2:**
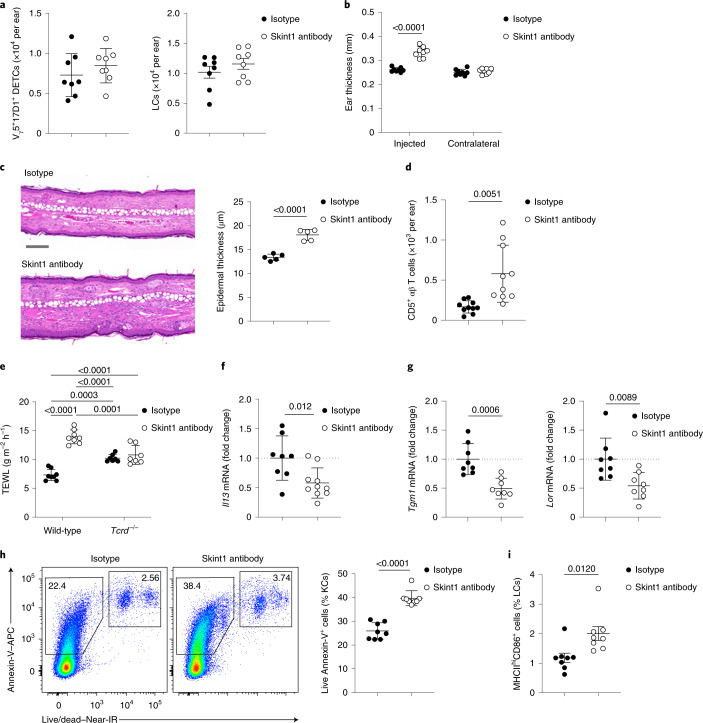
Skint1 underpins maintenance of epidermal integrity by DETCs. **a**, Flow cytometric quantification of V_γ_5V_δ_1^+^ DETCs and LCs in wild-type FVB mice administered Skint1 antibody or isotype control by i.d. injection into the ear weekly for 10 weeks (*n* = 8 per group). **b**, Total ear thickness at 10 weeks (*n* = 8 per group) in mice treated as in **a**. **c**, Hematoxylin and eosin (H&E) sections of injected ear skin and quantification of epidermal thickness at 5 weeks in mice treated as in **a** (*n* = 5 per group); scale bar, 100 μm. **d**, Flow cytometric quantification of epidermal CD3^+^CD5^+^TCRβ^+^ T cells in mice treated as in **a** (*n* = 10 per group). **e**, Transepidermal water loss from ear skin in wild-type (*n* = 8 per group) and *Tcrd*^–/–^ mice (*n* = 7 per group) administered Skint1 antibody or isotype control by i.d. injection into the ear weekly for 5 weeks. **f**, Quantitative PCR (qPCR) of *Il13* expression in the epidermis of wild-type mice treated as in **e** (*n* = 8 isotype control; *n* = 10 Skint1 antibody). **g**, qPCR of KC gene expression in the epidermis of mice treated as in **a** (*n* = 8 per group). **h**, Flow cytometric analysis of viability and apoptosis of CD45^–^ KCs in mice treated as in **a** (*n* = 8 per group); IR, infrared. **i**, Flow cytometry of activated LC frequency in mice treated as in **a** (*n* = 8 per group). Data are representative of (**a**–**c**, **h** and **i**) or are pooled from (**d**–**g**) at least two independent experiments per time point. A paired two-way ANOVA with Sidak’s multiple comparisons test (**b**), an unpaired two-sided *t*-test (**c**, **f**–**i**), an unpaired two-sided *t*-test with Welch’s correction (**d**) or an ordinary two-way ANOVA with Tukey’s multiple comparisons test (**e**) was used to analyze the data. Error bars represent mean ± s.d. Source data

**Extended Data Fig. 2:**
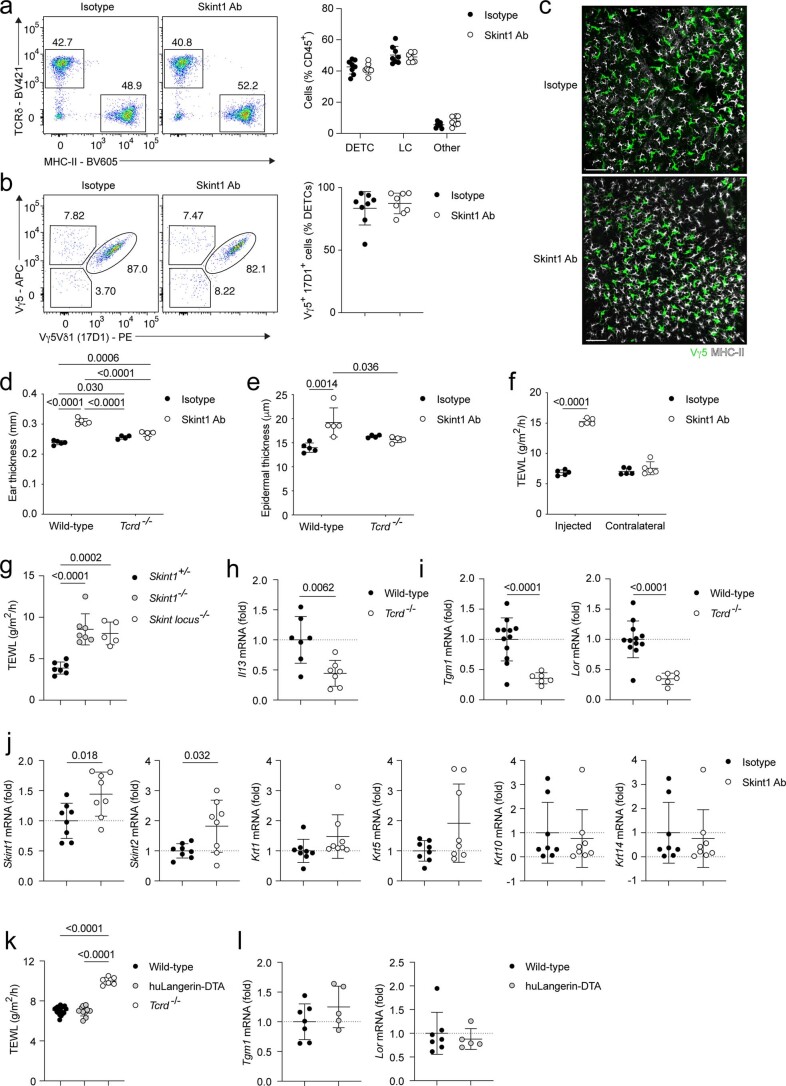
Skint1 underpins maintenance of epidermal integrity by DETCs. **a)** Flow cytometry of epidermal CD45^+^ cellular composition and **b)** canonical TCR expression by DETCs from wild-type FVB mice administered with Skint1 Ab or isotype control by intradermal injection into the ear weekly for 10 weeks (n=8/group). **c**) Confocal microscopy of Vγ5^+^ DETCs (green) and MHC-II^+^ LCs (white) from mice treated as in a. Representative of n=8/group, scale bar 50 μm. **d**) Total ear thickness of wild-type FVB (n=5/group) or *Tcrd*^*-/-*^ mice (n=4/group) administered with Skint1 Ab or isotype control by intradermal injection into the ear weekly for 5 weeks. **e)** Epidermal thickness measured from H&E sections of ear skin of mice treated as in d. **f**) Transepidermal water loss (TEWL) of injected and contralateral ear skin of wild-type mice treated as in d. **g**) Baseline TEWL in C57BL/6 *Skint1*^*+/-*^ (n=7), *Skint1*^*-/*-^ (n=7) and *Skint* locus^-/-^ mice (n=5). **h**) qPCR of *Il13* expression in epidermis of untreated wild-type FVB (n=14) and *Tcrd*^-/-^ mice (n=9). **i)** qPCR of keratinocyte gene expression in epidermis of untreated wild-type FVB (n=15) and *Tcrd*^-/-^ mice (n=9). **j)** qPCR of keratinocyte gene expression in epidermis of mice treated as in a (n=8/group). **k**) Baseline TEWL in wild-type FVB (n=18), Langerin-DTA (n=11) or *Tcrd*^*-/-*^ mice (n=6). **l**) qPCR of keratinocyte gene expression in epidermis of wild-type FVB (n=7) and Langerin-DTA mice (n=5). Data representative of at least 2 independent experiments (**a-b, d, f, k-l**) or pooled from 3 (**h-i**) 2 (**j**) independent experiments. Ordinary two-way ANOVA with Tukey’s multiple comparisons test (**d-e**), ordinary one-way ANOVA with Dunnett’s multiple comparisons test relative to *Skint1*^+/-^ (**g**), paired two-way ANOVA with Sidak’s multiple comparisons test (**f**), unpaired two-tailed t-test (**h-j, l**) or ordinary one-way ANOVA with Tukey’s multiple comparisons test (**k**). Error bars represent mean ± SD. Source data

To analyze the impact of sustained Skint1 disruption in situ, we performed an i.d. injection of Skint1 antibody or isotype control weekly into the ears of FVB mice for 10 weeks. At this time point, mice displayed comparable numbers and frequencies of V_γ_5V_δ_1^+^ DETCs and major histocompatibility complex class II^+^ (MHCII^+^) Langerhans cells (LCs) (Fig. 2a and Extended Data Fig. 2a–c), indicating that long-term DETC population maintenance was Skint1 independent. However, relative to isotype-treated or contralateral ears, Skint1 antibody-treated ears became moderately inflamed (Fig. 2b,c), and CD5^+^ αβ T cells infiltrated the epidermis (Fig. 2d). Similar increased ear thickness, albeit milder, was observed in control-treated *Tcrd*^–/–^ mice (Extended Data Fig. 2d), as previously reported^26^. Sustained Skint1 antibody treatment did not further increase ear thickness in *Tcrd*^–/–^ mice (Extended Data Fig. 2d,e), indicating that Skint1 antibody treatment primarily interfered with the Skint1–TCRγδ interaction.

We additionally observed increased transepidermal water loss (TEWL) at week 5 of Skint1 antibody treatment relative to isotype treatment (Fig. 2e) or contralateral ears (Extended Data Fig. 2f). Increased baseline TEWL measurements were also detected in isotype-treated *Tcrd*^–/–^ mice, as reported in ref. ^4^, and those were also unaffected by Skint1 antibody treatment (Fig. 2e). C57BL/6 mice lacking *Skint1* or all *Skint* genes also had elevated baseline TEWL compared to littermate controls (Extended Data Fig. 2g). Compared to isotype-treated mice, Skint1 antibody-treated mice consistently displayed decreased epidermal expression of the DETC-derived barrier maintenance cytokine *Il13* (ref. ^27^) and of KC genes involved in epidermal barrier formation (*Tgm1* and *Lor*; Fig. 2f,g), as did baseline *Tcrd*^–/–^ mice relative to wild-type controls (Extended Data Fig. 2h,i). KCs also displayed increased apoptosis following Skint1 antibody treatment (Fig. 2h). While keratin gene expression was unchanged, indicating normal KC differentiation, epidermal *Skint1* and *Skint2* expression was upregulated following Skint1 antibody treatment (Extended Data Fig. 2j), perhaps reflecting a compensatory response to Skint1–TCR disruption. Although LC abundance was unchanged (Fig. 2a and Extended Data Fig. 2a,c), Skint1 antibody treatment marginally increased the frequency of activated MHCII^hi^CD86^+^ LCs (Fig. 2i), perhaps reflecting ongoing epidermal inflammation. Most DETCs contact LCs at steady state, and LCs may also contribute to epidermal integrity^28,29^. However, we did not observe differences in baseline TEWL or barrier gene expression in LC-deficient human Langerin (huLangerin)-DTA mice relative to wild-type mice (Extended Data Fig. 2k,l). Thus, the functional contributions of DETCs to effective epidermal integrity were compromised by Skint1 blockade and were LC independent.

### Skint1 sensing maintains DETC immunosurveillance signatures

**Fig. 3:**
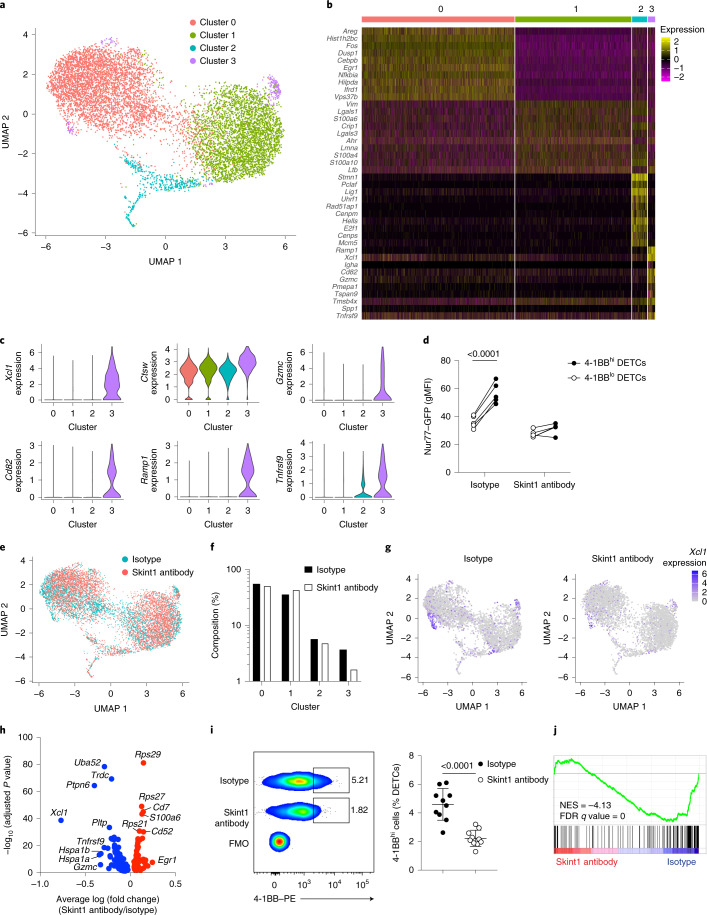
Peripheral Skint1 sustains steady-state DETC signatures of immunosurveillance. **a**, UMAP of the combined single-cell RNA-sequencing dataset of DETCs from wild-type FVB mice administered Skint1 antibody or isotype control by i.d. injection 1 week before (*n* = 1 per group). **b**, Top 10 most significantly enriched genes per cluster in DETCs from the combined dataset, as in **a**. **c**, Expression of key cluster 3 marker genes in the combined dataset, as in **a**. **d**, Flow cytometry expression of green fluorescent protein (GFP) in DETCs segregated by 4-1BB expression from Nur77–GFP mice treated 2 d prior with Skint1 antibody or isotype control by i.d. injection (*n* = 5 per group); gMFI, geometric mean fluorescence intensity. **e**, UMAP of DETCs comparing treatments, as in **a**. **f**, Contribution of DETCs from Skint1 antibody- and isotype-treated samples to cluster composition, as in **a**. **g**, Expression of *Xcl1* in DETCs, comparing Skint1 antibody and isotype treatments, as in **a**. **h**, Differential gene expression in combined DETC clusters comparing Skint1 antibody and isotype treatments, as in **a**; FMO, fluorescence minus one **i**, Frequency of 4-1BB^hi^ DETCs by flow cytometry in wild-type FVB mice treated with Skint1 antibody or isotype control by i.d. injection 1 week prior (*n* = 10 per group). **j**, Gene set enrichment analysis (GSEA) of combined DETC clusters comparing Skint1 antibody and isotype treatments, as in **a**, using a Skint1 selection gene signature from DETC precursors isolated from wild-type or FVB.Tac embryonic thymi; NES, normalized enrichment score; FDR, false discovery rate. Data are representative of three independent experiments (**d**) or are pooled from two independent experiments (**i**). A Wilcoxon test with Bonferroni’s correction (**b** and **h**), paired one-way ANOVA with Sidak’s multiple comparisons test (**d**) or unpaired two-sided *t*-test (**i**) was used to analyze the data. Error bars represent mean ± s.d. Source data

To investigate whether the contribution of Skint1 to skin physiology reflected downstream consequences of a primary impact on DETCs, we conducted single-cell RNA sequencing of CD45^+^MHCII^−^EpCAM^–^ epidermal lymphocytes from wild-type FVB mice 7 d after Skint1 antibody or isotype treatment. Non-DETC populations, including αβ T cells and contaminating dermal γδ T cells, were excluded. Four DETC clusters (cluster 0–cluster 3) were identified by uniform manifold projection (UMAP) of the integrated dataset (Fig. 3a), indicating that DETCs were heterogeneous, despite their near uniform anatomical localization and TCR expression. Cluster 3 appeared segregated in two dimensions (Fig. 3a), an artifact of dimensionality reduction that could not be resolved by adjusting algorithm parameters.

**Extended Data Fig. 3:**
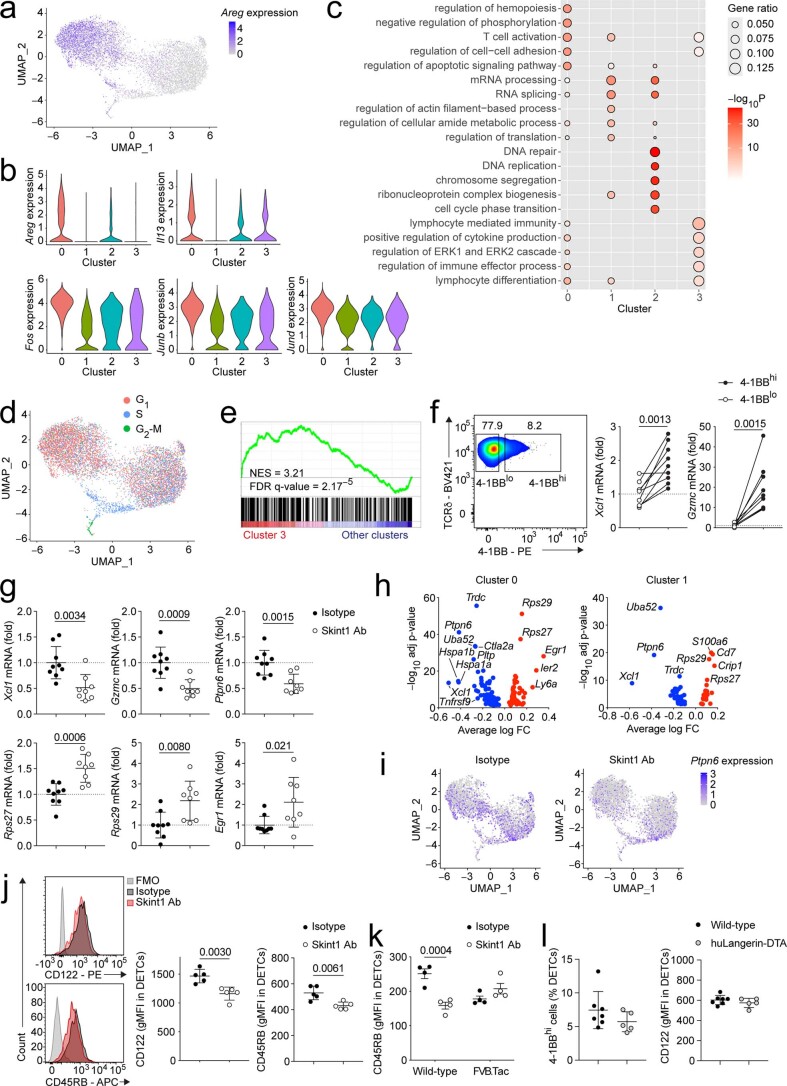
Peripheral Skint1 sustains steady-state DETC signatures of immunosurveillance. **a)** Expression of *Areg* in DETCs from the combined dataset of single cell RNA sequencing of DETCs from wild-type FVB mice treated 7d prior with Skint1 Ab or isotype control by intradermal injection (n=1/group). **b)** Expression of selected Cluster 0 marker genes from the combined dataset as in a. **c)** Selected enriched gene ontology terms in each DETC cluster from the combined dataset as in a. **d)** Seurat cell cycle analysis of DETCs from the combined dataset as in a. **e)** Gene set enrichment analysis (GSEA) comparing Cluster 3 with other clusters in the combined dataset as in a, using publicly available gene set “GO positive regulation of immune response”. **f)** qPCR for cluster 3 marker gene expression within sorted 4-1BB^hi^ and 4-1BB^lo^ DETCs from untreated wild-type FVB mice (n=9). **g)** qPCR validation of differentially expressed genes in total DETC sorted from mice treated as in a (isotype n=9, Skint1 Ab n=8). **h)** Differentially expressed genes in Clusters 0 and 1 comparing Skint1 Ab and isotype treatments as in a. **i)** Expression of *Ptpn6* in DETCs comparing Skint1 Ab and isotype treatments as in a. **j)** Flow cytometry of CD122 and CD45RB expression by DETCs from mice treated as in a (n=5/group). **k**) Flow cytometry of CD45RB expression by TCRγδ^+^ DETCs from wild-type FVB and FVB.Tac mice treated as in a (n=4/group). **l**) Flow cytometry of 4-1BB^hi^ DETCs and CD122 expression by DETCs from wild-type FVB (n=7) and Langerin-DTA mice (n=5). Pooled data from two experiments (**f-g**) or representative of at least two experiments (**j, l**). Hypergeometric test with Benjamini-Hochberg correction (**c**), paired two-tailed t-test (**f**), unpaired two-tailed t-test (**g, j, l**), Wilcoxon test with Bonferroni’s correction (**h**), ordinary two-way ANOVA with Sidak’s multiple comparisons test (**k**). Error bars represent mean ± SD. Source data

Cluster 0 was enriched in transcripts related to AP1-mediated cell regulation (*Fos*, *Junb* and *Jund*) and the epithelia-tropic cytokines *Areg* (amphiregulin) and *Il13* (Fig. 3b and Extended Data Fig. 3a–c), implying a potential to regulate KCs and other immunocytes^27,30–32^. Relative to other clusters, cluster 1 appeared more quiescent, with reduced expression of gene sets associated with T cell activation, immune effector processes and regulation of cell adhesion and apoptosis (Extended Data Fig. 3b,c). Nonetheless, cluster 1 was enriched in transcripts encoding galectins, S100 proteins and the aryl hydrocarbon receptor (*Ahr*) (Fig. 3b), of which the latter is essential for DETC population self-renewal^33^. Germane to that, cluster 2 was enriched in transcripts associated with proliferation (Fig. 3b and Extended Data Fig. 3c,d). Cluster 3, while small (Fig. 3a), was enriched in mRNAs ascribed to lymphocyte activation and costimulation, such as *Cd82*, *Ramp1* and *Tnfrsf9* (4-1BB), as well as effector molecules *Xcl1*, *Ctsw*, *Gzmc* (granzyme C) and *Il13* (Fig. 3b,c and Extended Data Fig. 3b,c,e). 4-1BB is conventionally viewed as a costimulatory receptor expressed by recently activated T cells, but it is also induced by Skint1- and Btnl1-mediated selection of DETCs and gut V_γ_7^+^ IEL progenitors, respectively^15,34,35^. Indeed, flow cytometry sorting indicated that 4-1BB^hi^ DETCs expressed more *Gzmc* and *Xcl1* than 4-1BB^lo^ DETCs (Extended Data Fig. 3f). In Nur77–GFP reporter mice, which report calcium-dependent signaling following T cell activation^36^, GFP expression was significantly higher in 4-1BB^hi^ DETCs at steady state (Fig. 3d). These results suggested that cluster 3 might represent a subset of DETCs actively responding to minor skin perturbations, such as grooming or scratching, which occur episodically in mice at steady state.

Comparison of DETCs from isotype- and Skint1 antibody-treated mice indicated that cluster 3 was selectively diminished by Skint1 antibody treatment (Fig. 3e,f), which was reflected by a loss of overall *Xcl1* and *Gzmc* expression (Fig. 3g,h) and validated by qPCR of DETCs sorted from Skint1 antibody- and isotype-treated mice (Extended Data Fig. 3g). Consistently, 4-1BB^hi^ DETCs were significantly reduced by Skint1 antibody treatment (Fig. 3i), and the remaining 4-1BB^hi^ DETCs expressed less Nur77–GFP (Fig. 3d). Thus, sustained activation of a small subset of DETCs (cluster 3) requires Skint1 sensing at steady state.

We also observed broader changes in gene expression in DETCs following Skint1 antibody treatment, regardless of clusters. *Ptpn6* (SHP-1 phosphatase) and overall *Xcl1* expression decreased, and *Rps27*, *Rps29* and *Egr1* expression increased following Skint1 antibody treatment (Fig. 3h and Extended Data Fig. 3h), as validated by qPCR (Extended Data Fig. 3g). This indicated that the intrinsic activation state of most DETCs depended on sustained Skint1-dependent DETC–KC interactions. Peripheral Skint1-dependent regulation of *Ptpn6* expression in most DETCs (Extended Data Fig. 3i) was reminiscent of the upregulation of *Ptpn6* during Skint1-dependent DETC precursor selection (Supplementary Table 1). Similarly, Skint1 antibody treatment moderately reduced DETC expression of CD122 and CD45RB proteins (Extended Data Fig. 3j), which are also upregulated during BTNL-dependent IEL selection^15,16^. Skint1 antibody-treated wild-type DETCs resembled non-V_γ_5V_δ_1^+^ DETCs in FVB.Tac mice regarding CD45RB expression (Extended Data Fig. 3k). GSEA indicated that the collective gene signature of thymic Skint1-driven DETC progenitor selection^35^ was downregulated in mature DETC by Skint1 antibody treatment (Fig. 3j and Supplementary Table 1). 4-1BB^hi^ DETC frequency and CD122 expression were unaltered in huLangerin-DTA mice (Extended Data Fig. 3l), suggesting that Skint1 regulation of DETCs was direct and LC independent. Thus, steady-state epidermal Skint1 promoted immunosurveillance by maintaining the predetermined effector phenotype in most DETCs and by underpinning the activation of a small subset of DETCs.

### Skint1 sensing drives DETC responses to UVR

**Fig. 4:**
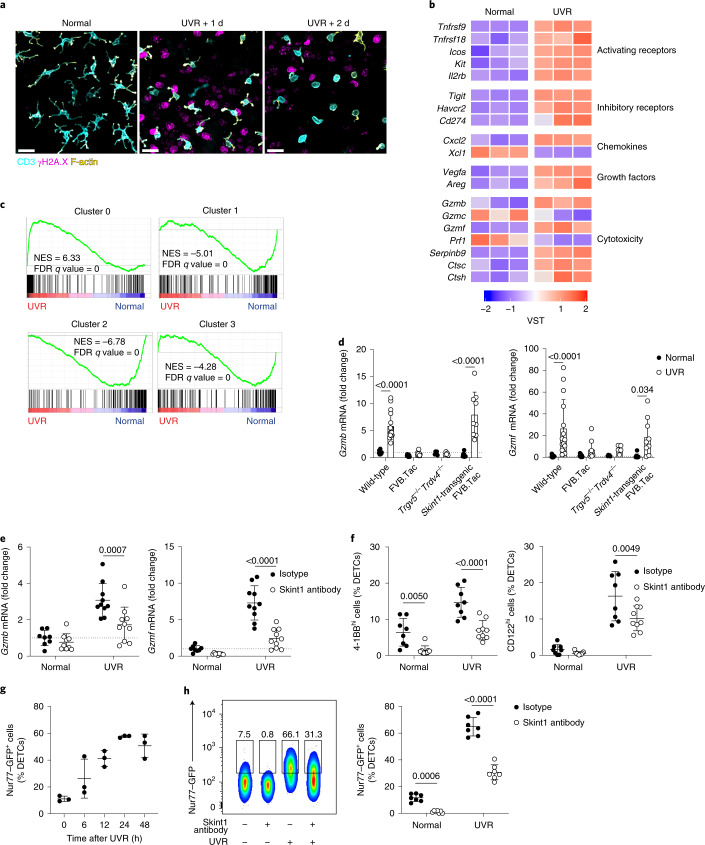
DETC sensing of genotoxic epithelial stress requires acute exposure to Skint1. **a**, Confocal imaging of CD3^+^ DETCs (cyan), F-actin within a CD3^+^ mask (yellow) and γH2A.X^+^ nuclei (magenta) following UVR of ear skin of wild-type C57BL/6 mice. Data are representative of *n* = 5 per time point; scale bar, 20 μm. **b**, Scaled gene expression from RNA sequencing of DETCs isolated from ear skin 24 h after UVR of wild-type C57BL/6 mice (*n* = 3 per group); VST, variance-stabilizing transformation. **c**, GSEA of UVR-induced gene expression changes in DETCs using signatures of steady-state DETC clusters from Fig. 3. **d**, qPCR for DETC gene expression in the epidermis of FVB wild-type (*n* = 15), Tac (*n* = 9), *Trgv5*^–/–^*Trdv4*^–/–^ (*n* = 5) and *Skint1*-transgenic FVB.Tac (*n* = 9) mice 24 h after UVR. **e**, qPCR for effector genes in DETCs sorted at day 1 after UVR from wild-type C57BL/6 mice pretreated with i.d. injection of Skint1 antibody or isotype control (isotype control normal, *n* = 8; Skint1 antibody normal, *n* = 9; isotype control/Skint1 antibody UVR, *n* = 10 per group). **f**, Flow cytometry of 4-1BB and CD122 expression in DETCs from wild-type C57BL/6 mice treated as in **e** (*n* = 8 isotype control; *n* = 9 Skint1 antibody). **g**, Flow cytometry of GFP expression in DETCs following UVR of Nur77–GFP reporter mice (*n* = 3 per time point). **h**, Flow cytometry of GFP expression in DETCs from Nur77–GFP reporter mice treated as in **e** (*n* = 7 per group). Data are pooled data from two (**e** and **f**) or three (**d**) independent experiments or are representative of two (**a**) or three (**h**) independent experiments. A paired (**d**, **f** and **h**) or ordinary (**e**) two-way ANOVA with Sidak’s multiple comparisons test was performed. Error bars represent mean ± s.d. Source data

**Extended Data Fig. 4:**
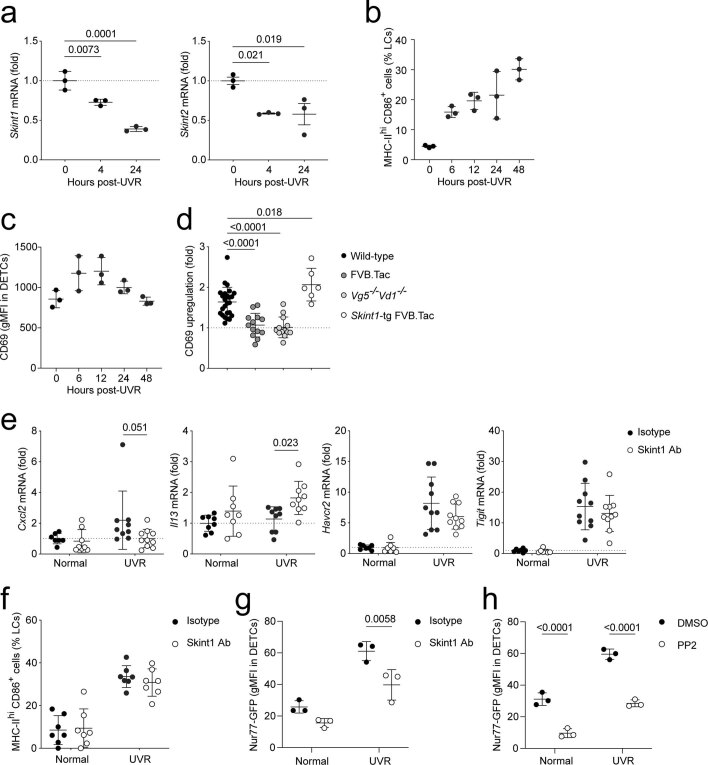
DETC sensing of genotoxic epithelial stress requires acute exposure to Skint1. **a)** qPCR expression of *Skint1* and *Skint2* in KCs sorted from wild-type C57BL/6 mice at indicated timepoints following UVR (n=3/timepoint). **b**) Flow cytometry frequency of activated LCs following UVR of ear skin in wild-type C57BL/6 mice (n=3/timepoint). **c**) Flow cytometry of CD69 expression by DETCs following UVR of ear skin in wild-type C57BL/6 mice (n=3/timepoint). **d**) Flow cytometry analysis of fold change in CD69 gMFI on DETCs 24hr post-UVR relative to unchallenged skin from wild-type FVB (n=25), FVB.Tac (n=13), Trgv5^-/-^Trdv4^-/-^ (n=13) and *Skint1*-transgenic FVB.Tac mice (n=6). **e)** qPCR for effector genes in DETCs sorted from wild-type C57BL/6 mice at 24hr post-UVR, following pre-treatment with intradermal injection of Skint1 Ab or isotype control (isotype normal n=8, Skint1 Ab normal n=8, isotype UVR n=10, Skint1 Ab UVR n=10). **f**) Flow cytometry frequency of activated LCs from mice treated as in e) (n=7/group). Flow cytometry of GFP expression by DETCs from ear sheets of Nur77-GFP mice which were cultured for 4hr in medium containing **g)** anti-Skint1 or isotype control antibodies and **h)** PP2 or DMSO vehicle prior to UVR, and analyzed 24hr later (n=3/group). Pooled data from 4 (**d**) or 2 (**e**) independent experiments, or representative of 3 experiments (**g-h**). Ordinary one-way ANOVA with Dunnett’s multiple comparisons test relative to 0hr (**a**) or wild-type (**d**), paired two-way ANOVA with Sidak’s multiple comparisons test (**e, f-h**). Error bars represent mean ± SD. Source data

To further address the contribution of Skint1 to immunosurveillance, we irradiated the ears of C57BL/6 mice with UVR to induce DNA damage. Within 1 d, many KCs exhibited signs of DNA damage and repair, as measured by γH2A.X foci (Fig. 4a). Simultaneously, DETCs became rounded (Fig. 4a), consistent with reports of their response to diverse skin perturbations, including UVR^37–39^. We also observed a substantial diminution in juxtaepithelial TCR focusing (Fig. 4a) and *Skint1* and *Skint2* downregulation in KCs relative to unchallenged mice (Extended Data Fig. 4a). Concurrently, LCs and DETCs displayed upregulation of surface activation markers (Extended Data Fig. 4b,c).

RNA-sequencing analysis of flow cytometry-sorted DETCs 24 h after UVR revealed rapid and pleiotropic changes in gene expression compared to non-irradiated controls (Fig. 4b). This included further upregulation of *Tnfrsf9* (4-1BB) and *Il2rb* (CD122), upregulation of *Icos* and *Kit* (c-Kit), which encode activating/renewal receptors, and upregulation of inhibitory receptors *Tigit*, *Havcr2* (TIM-3) and *Cd274* (PD-L1) (Fig. 4b). Moreover, UVR resulted in upregulation of many genes encoding potential effector molecules, including *Cxcl2*, *Vegfa*, *Gzmb*, *Gzmf, Ctsc* and *Ctsh* (Fig. 4b). GSEA revealed an enrichment in a cluster 0 signature in DETCs following UVR (Fig. 4c), indicating that DETCs further upregulate cluster 0-defining genes following UVR activation and/or that DETCs in other clusters assume a cluster 0 phenotype. Notably, DETCs from irradiated FVB.Tac and *Trgv5*^–/–^*Trdv4*^–/–^ mice did not display upregulation of *Gzmb*, *Gzmf* or CD69 compared with wild-type FVB and *Skint1*-transgenic FVB.Tac mice (Fig. 4d and Extended Data Fig. 4d), strongly suggesting that the canonical V_γ_5V_δ_1 TCR was essential for UVR responsiveness.

To test whether Skint1-dependent TCR–KC interactions established and/or maintained the preparedness of DETCs to respond to UVR, Skint1 antibody was injected i.d. into wild-type C57BL/6 mice 1 d before UVR, and DETCs were sorted and analyzed by qPCR 24 h after UVR. The UVR-induced upregulation of *Gzmb*, *Gzmf* and *Cxcl2* was significantly reduced in the Skint1 antibody-treated mice, while UVR-induced inhibitory receptor genes, such as *Havcr2* and *Tigit*, remained unaffected, and *Il13* was atypically upregulated compared to isotype-treated mice (Fig. 4e and Extended Data Fig. 4e). In addition, the UVR-induced increase in 4-1BB and CD122 expression was significantly inhibited by prior Skint1 antibody administration (Fig. 4f), while LC activation was unperturbed (Extended Data Fig. 4f). In Nur77–GFP reporter mice, DETCs displayed a basal level of GFP, which increased incrementally following UVR (Fig. 4g). Skint1 antibody treatment not only diminished the basal Nur77–GFP signal in DETCs but substantially suppressed the UVR-induced increase in Nur77–GFP expression (Fig. 4h). These observations were reproduced ex vivo in an explant model, in which DETCs in Nur77–GFP ear sheets preincubated with Skint1 antibody showed impaired upregulation of Nur77–GFP expression following in vitro exposure to UVR relative to isotype control (Extended Data Fig. 4g), indicating that Skint1 antibody acted locally. Pretreatment of ex vivo ear sheets with the Src-family kinase inhibitor PP2 produced a similar outcome (Extended Data Fig. 4h). Collectively, these observations indicate that Skint1-dependent interactions licensed DETC stress responsiveness and effector functions.

### Skint1 licenses DETC responsiveness to UVR via TNF receptors

**Fig. 5:**
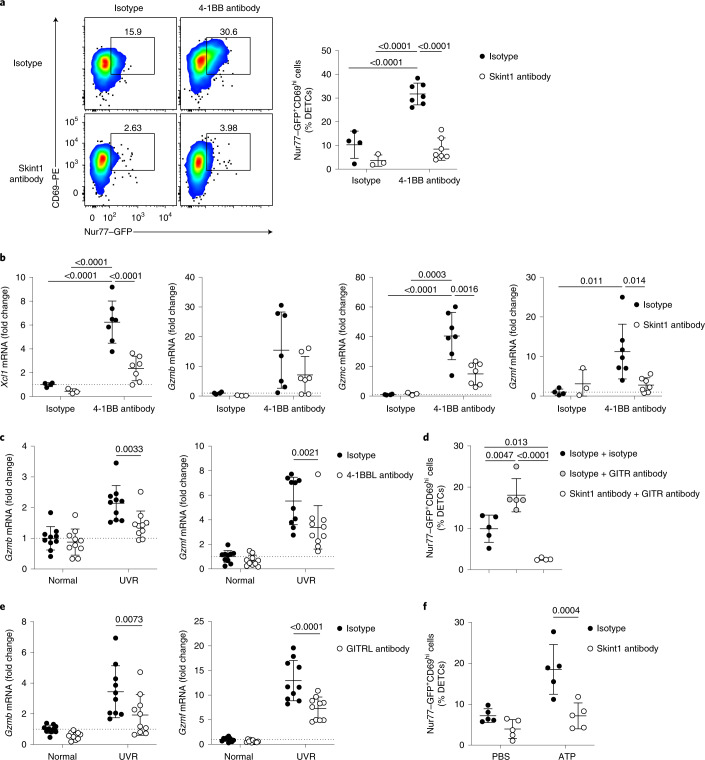
DETC stress surveillance via TNF superfamily receptors is licensed by Skint1. **a**,**b**, Flow cytometry (**a**) of DETC CD69 and GFP expression and qPCR (**b**) for DETC gene expression in the epidermis from Nur77–GFP reporter mice administered agonistic 4-1BB antibody or isotype control 24 h following Skint1 antibody or isotype control administration by i.d. injection and analyzed 6 h later (*n* = 4 isotype/isotype; *n* = 3 Skint1 antibody/isotype; *n* = 7 isotype/4-1BB antibody; *n* = 7 Skint1 antibody/4-1BB antibody). **c**, qPCR for DETC gene expression in the epidermis at 24 h after UVR from wild-type C57BL/6 mice pretreated with 4-1BBL antibody or isotype control by i.d. injection (*n* = 10 per group). **d**, Flow cytometry of GFP and CD69 expression in DETCs from Nur77–GFP mice administered agonistic glucocorticoid-induced TNF receptor-related protein (GITR) antibody or isotype control 24 h after i.d. injection with Skint1 antibody or isotype control and analyzed 6 h later (*n* = 5 isotype/isotype; *n* = 5 isotype/GITR antibody; *n* = 4 Skint1 antibody/GITR antibody). **e**, qPCR of DETC gene expression in the epidermis at 24 h after UVR from wild-type C57BL/6 mice pretreated with GITRL antibody or isotype control by i.d. injection (*n* = 10 per group). **f**, Flow cytometry of GFP and CD69 expression in DETCs from Nur77–GFP mice administered ATP or PBS 24 h after i.d. injection with Skint1 antibody or isotype control and analyzed 9 h later (*n* = 5 per group). Data are pooled from two independent experiments (**a**–**f**). An ordinary (**a** and **b**) or paired (**c**, **e** and **f**) two-way ANOVA with Sidak’s multiple comparisons test was performed or an ordinary one-way ANOVA with Tukey’s multiple comparisons test (**d**). Error bars represent mean ± s.d. Source data

**Extended Data Fig. 5:**
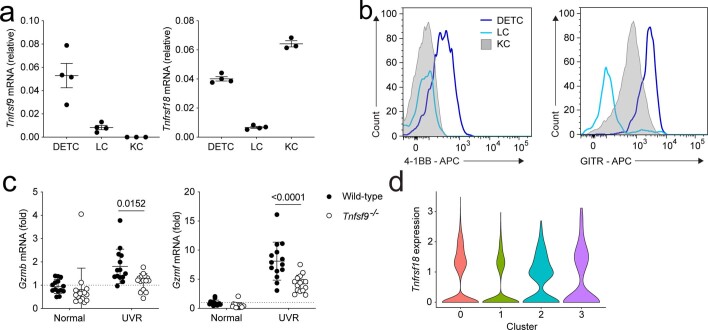
DETC stress surveillance via TNF superfamily receptors is licensed by Skint1. **a)** qPCR for gene expression (DETCs/LCs n=4, KCs n=3) and **b)** flow cytometry for surface expression of 4-1BB and GITR in epidermal cell populations from unchallenged wild-type C57BL/6 mice. Representative of n=3 mice (**b**). **c)** qPCR for DETC gene expression in epidermis at 24hr post-UVR from wild-type C57BL/6 (n=14) and *Tnfsf9*^*-/-*^ mice (n=15). **d)** *Tnfrsf18* expression in steady state DETC clusters identified from scRNAseq as in Fig. 3. Pooled data from 3 independent experiments (**c**). Paired two-way ANOVA with Sidak’s multiple comparisons test (**c**). Error bars represent mean ± SD. Source data

TCR signaling amplification is a defining property of costimulatory molecules, including those of the TNF receptor superfamily. To investigate whether steady-state Skint1-dependent interactions licensed DETCs to respond rapidly through co-receptors such as 4-1BB, we performed i.d. injections of an agonistic 4-1BB antibody or isotype control in the ears of Nur77–GFP mice. Akin to UVR, 4-1BB antibody drove a coordinated upregulation of CD69 and Nur77–GFP by a subset of DETCs relative to isotype control (Fig. 5a). This effect was likely direct, as epidermal 4-1BB expression was exclusive to DETCs (Extended Data Fig. 5a,b); however, i.d. injection with Skint1 antibody 24 h before 4-1BB stimulation ablated CD69 and Nur77–GFP upregulation in DETCs (Fig. 5a). Additionally, 4-1BB antibody injection induced expression of DETC effector genes related to cluster 3 (*Xcl1* and *Gzmc*) and UVR responses (*Gzmb* and *Gzmf*), which was similarly inhibited by prior Skint1 antibody treatment (Fig. 5b). These observations indicated that steady-state TCR–Skint1 interactions with KCs licensed DETCs to respond to 4-1BB, inducing qualitative changes that partially phenocopied the UVR response.

Therefore, to test whether 4-1BB was an obligate contributor to UVR responses, we performed an i.d. injection of an antibody that blocks 4-1BB ligand (4-1BBL) binding to 4-1BB into the ears of wild-type C57BL/6 mice before UVR treatment. This antibody partially, but significantly, blocked the UVR-induced increase in epidermal expression of *Gzmb* and *Gzmf* relative to isotype control (Fig. 5c). Consistent with this, UVR-induced upregulation of *Gzmb* and *Gzmf* was also inhibited in 4-1BBL-deficient *Tnfsf9*^–/–^ mice relative to wild-type controls (Extended Data Fig. 5c).

Similar to 4-1BB, expression of *Tnfrsf18* (which encodes GITR) was also induced by Skint1 during thymic DETC progenitor selection (Supplementary Table 2). Unlike 4-1BB, *Tnfrsf18* was not enriched in cluster 3 but was expressed by most DETCs (Extended Data Fig. 5d). Additionally, GITR mRNA and protein were expressed by KCs and a small fraction of LCs (Extended Data Fig. 5a,b). Regardless, i.d. injection of an agonistic GITR antibody also upregulated Nur77–GFP and CD69 expression by DETCs, which was almost entirely Skint1 dependent (Fig. 5d). Furthermore, antagonist antibodies that blocked GITR ligand (GITRL) interactions with GITR partially but significantly inhibited the UVR-induced upregulation of epidermal *Gzmb* and *Gzmf* (Fig. 5e). Thus, signaling through TNF receptors was one non-redundant component of the rapid DETC response to skin irradiation and was dependent on prior licensing by Skint1–TCR interactions.

Next, we asked whether other conduits of innate T cell activation required licensing via Skint1–TCR interactions. Extracellular ATP is an alarmin implicated in DETC responses to UVR together with interleukin-1 (IL-1)^37^. Administration (i.d.) of ATP alone induced Nur77–GFP upregulation in DETCs, which was Skint1 dependent (Fig. 5f). Thus, steady-state Skint1 sensing regulated the ability of DETCs to respond to diverse stimuli.

### Skint1-licensed immunosurveillance promotes recovery from epithelial damage

**Fig. 6:**
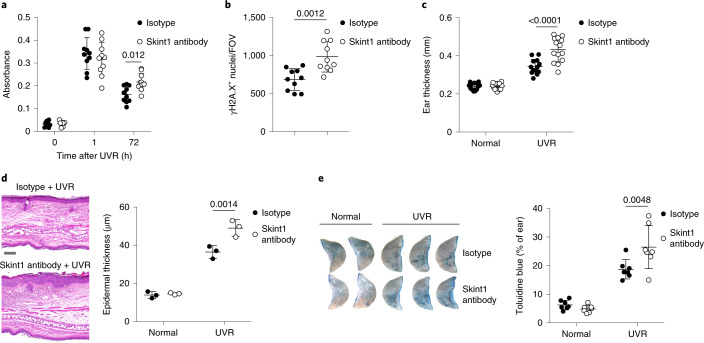
Skint1-dependent DETC responses promote epithelial recovery from DNA damage. **a**, Enzyme-linked immunosorbent assay (ELISA) for CPDs in epidermal DNA extracts from wild-type C57BL/6 mice administered Skint1 antibody or isotype control by i.d. injection 24 h before and 48 h after UVR of dorsal ear skin (*n* = 10 per group). **b**, Confocal microscopy quantification of γH2A.X nuclei in ear sheets per field of view (FOV) at day 3 after UVR in mice treated as in **a** (*n* = 10 per group; mean value reported per mouse). **c**, Total ear thickness at day 5 after UVR compared to that observed in contralateral unirradiated ears in mice treated as in **a** (isotype, *n* = 13; Skint1 antibody, *n* = 14). **d**, H&E histology of ear sections and quantification of mean epidermal thickness at day 5 after UVR compared to the non-irradiated ventral side in mice treated as in **a**; scale bar, 100 μm (*n* = 3 per group). **e**, Toluidine blue permeation of ears at day 5 after UVR in mice treated as in **a** (*n* = 7 per group). Data are pooled from two (**a**, **b** and **e**) or three (**c**) independent experiments or are representative of two independent experiments (**d**). Paired two-way ANOVA with Sidak’s multiple comparisons tests (**a** and **c**–**e**) or an unpaired two-sided *t*-test (**b**) was used to analyze the data. Error bars represent mean ± s.d. Source data

**Extended Data Fig. 6:**
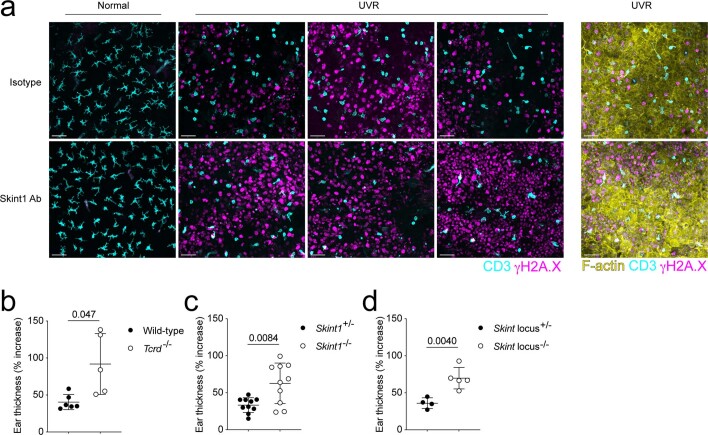
Skint1-dependent DETC responses promote epithelial recovery from DNA damage. **a)** Wild-type C57BL/6 mice were administered Skint1 Ab or isotype control by intradermal injection 24hr prior to and 48hr post-ultraviolet radiation (UVR) of dorsal ear skin. Representative confocal microscopy images of γH2A.X nuclei (magenta) and CD3^+^ DETCs (cyan) in ear sheets, as quantified in Fig. 6b at d3 post-UVR. Righthand images overlay F-actin staining (yellow). Scale bar 50μm (n=10/group). Relative increase in ear thickness at 5d post-UVR relative to unchallenged ear in **b)** wild-type C57BL/6 (n=6) vs *Tcrd*^-/-^ mice (n=5), **c)** littermate control (n=10) vs *Skint1*^-/-^ mice (n=10) and **d)** littermate control (n=4) vs *Skint* locus^-/-^ mice (n=5). Representative of (**a**) or pooled data from (**c**) 2 independent experiments. Unpaired two-tailed t-test (**b-d**) with Welch’s correction where necessary (**b-c**). Error bars represent mean ± SD. Source data

To investigate the consequences of Skint1 blockade for host physiology, we performed an i.d. injection of Skint1 antibody or isotype control 24 h before and 48 h after UVR. Mutagenic cyclobutane pyrimidine dimers (CPDs) were rapidly induced by UVR in all mice but were removed less efficiently in Skint1 antibody-treated mice than in isotype control-treated mice 72 h after UVR (Fig. 6a). Likewise, higher numbers of γH2A.X foci, more ear swelling and greater epidermal thickening were detected in Skint1 antibody-treated mice (Fig. 6b–d and Extended Data Fig. 6a). Exacerbated ear swelling was similarly observed after UVR in C57BL/6 mice deficient in γδ T cells, *Skint1* and the *Skint* locus (Extended Data Fig. 6b–d), providing genetic evidence that the pathophysiologic impacts of acute Skint1 blockade reflected consequences of disrupted Skint1-dependent TCR–KC interactions. Finally, UVR-associated defects in barrier function, as measured by toluidine blue permeation^40^, were also significantly exacerbated in Skint1 antibody-treated mice (Fig. 6e).

**Fig. 7:**
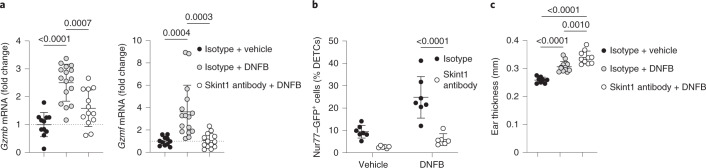
Skint1 licenses DETC responses to irritant contact dermatitis. **a**, qPCR for effector gene expression in sorted DETCs at 48 h after treatment from wild-type C57BL/6 mice administered isotype control or Skint1 antibody by i.d. injection before treatment of ear skin with 0.5% DNFB solution or vehicle (isotype/isotype, *n* = 11; isotype/DNFB, *n* = 16; Skint1 antibody/DNFB, *n* = 13). **b**, Flow cytometry of GFP expression by DETCs from Nur77–GFP reporter mice at 48 h after treatment as in **a** (isotype, *n* = 7; Skint1 antibody, *n* = 6). **c**, Total ear thickness at 48 h after treatment as in **a** (isotype/isotype, *n* = 8; isotype/DNFB, *n* = 12; Skint1 antibody/DNFB, *n* = 10). Data are pooled from two (**b** and **c**) or three (**a**) experiments. An ordinary one-way ANOVA with Tukey’s multiple comparisons test (**a** and **c**) or paired two-way ANOVA with Sidak’s multiple comparisons test (**b**) was used to analyze the data. Error bars represent mean ± s.d. Source data

Skin is commonly exposed to non-mutagenic chemical irritants. To investigate whether Skint1 licensing applied in contexts other than DNA damage, we examined a model of irritant contact dermatitis by using a single topical dose of the hapten dinitrofluorobenzene (DNFB). DETCs upregulated *Gzmb* and *Gzmf* within 48 h after DNFB application, but this was largely inhibited by prior Skint1 antibody treatment (Fig. 7a). Similarly, Skint1 blockade suppressed the DNFB-dependent induction of Nur77–GFP in DETCs (Fig. 7b). Moreover, acute DNFB-induced dermatitis was amplified in mice treated with Skint1 antibody (Fig. 7c). Thus, Skint1 licensed prompt DETC effector responses in settings of mutagenic and non-mutagenic epidermal stress, naturally limiting inflammatory lesions and impairments in functional barrier integrity.

## Discussion

Here we showed that steady-state V_γ_5V_δ_1^+^ TCR-mediated interactions between DETCs and KCs involve and depend on Skint1. This crosstalk did not obviously regulate the tissue residency of DETCs nor was it required for DETCs to populate the epidermis. However, it was essential for DETC function, as disruption of Skint1 sensing impeded DETC maintenance of epidermal homeostasis, interfered with steady-state immunosurveillance signatures, and compromised efficient DETC responsiveness to epithelial stresses. Indeed, TCR-dependent, Skint1-dependent interactions with KCs licensed DETCs for innate-like responsiveness through TNF receptors, which limited tissue pathology following insults that included UVR and a chemical irritant.

Because Skint1 is expressed by differentiated suprabasal epithelial cells and is stress-labile, DETC antigen receptors sense normality. This contrasts with the conventional consideration that TCRs engage products of change, such as microbial- or cancer-associated neoantigens and stress antigens upregulated by cellular dysregulation. It is possible that the DETC TCR also transduces signals from an unelucidated stress-induced ligand distinct from Skint1 (ref. ^41^). Nonetheless, stress sensing by DETCs depended largely on innate receptors 4-1BB and GITR, drawing parallels to NKG2D-driven DETC surveillance^12^. Critically, these receptors were acquired during thymic Skint1-dependent DETC progenitor selection^35^ and were sustained in the periphery by ongoing Skint1–TCR-mediated interactions. In this way, among others, steady-state TCR-mediated interactions licensed DETCs to respond rapidly to gross insults.

There are several major consequences of TCR-mediated normality sensing. First, Skint1-dependent, TCR-mediated interactions regulated barrier function and tissue homeostasis, which may occur by regulation of KC-tropic growth factor expression by DETCs. While IL-13 contributes to inflammatory pathologies^42^, steady-state IL-13 production by DETCs reportedly benefits epidermal integrity^27^. Although mice lacking V_γ_5V_δ_1^+^ DETCs do not suffer life-threatening barrier deficiencies, they do display cutaneous inflammation that could reflect compromised barrier function^4,16^, as we observed following sustained Skint1 blockade.

Second, Skint1 sustained the signature phenotype of mature DETCs, largely recapitulating its central role in DETC progenitor selection^16,17,24,35^. This suggests that while the effector potential of DETCs, including predetermined expression of TNFRs and other receptors, is programmed by thymic Skint1 selection, maintaining this profile requires continued peripheral Skint1 sensing. Cluster 3 DETCs, displaying hallmarks of recent activation in unchallenged mice, could represent Skint1-licensed natural immunosurveillance responses to spontaneous epithelial perturbations from scratching/grooming or mutagenesis. Intriguingly, DETC-derived XCL1 reportedly recruits cross-presenting dendritic cells (DCs) to the epidermis^43^. Thus, XCL1 release from cluster 3 DETCs responding to episodic, low-level KC dysregulation might, in cases of infection or genotoxicity, facilitate neoantigen uptake by DCs, thereby initiating cytotoxic CD8^+^ T cell immunosurveillance.

Third, Skint1 blockade reduced the capacity of DETCs to sense tissue perturbation through innate receptors. In the context of UVR, the prime etiologic agent of human skin cancer, we observed sustained genotoxicity and inflammatory pathology, which may in part explain the regulation of cutaneous carcinogenesis by DETCs and analogous roles fulfilled by other mouse and human γδ T cell compartments^11,44^. Additionally, normality sensing primed DETC responses to an irritant and to ATP, and it is conceivable that it might prime for wound healing and tissue regeneration. Indeed, disruption of TCR signaling in mature DETCs impaired wound healing^45^, a process reportedly modulated by Skint family members other than Skint1 and Skint2 (ref. ^46^).

Normality sensing may license invariant DETCs to respond through a panoply of innate receptors that collectively offer versatility to detect myriad manifestations of tissue perturbations with which IELs have been associated. Tissue-wide DNA damage, like that induced by UVR, resulted in overt granzyme upregulation, which may facilitate DETC killing of potentially malignant KCs^47^. Granzyme B also cleaves extracellular targets, including junctional molecules, surface receptors and extracellular matrix, whereas granzyme F is cytotoxic in vitro^48,49^. Other aspects of the DETC UVR response, including rounding up and TIM-3 upregulation, did not obviously require Skint1 licensing. Possibly, licensing regulates those traits that are inherently more dangerous if precociously expressed.

Overall, our results provide experimental evidence supporting the lymphoid stress surveillance hypothesis, according to which lymphocytes display myelomonocytic traits, such as responding rapidly through innate receptors^10^. However, the results also establish the need for prior licensing through the TCR. Whereas there is no direct DETC equivalent in humans, this concept may pertain to the selection and regulation of human intestinal innate-like V_γ_4^+^ IELs by BTNL3 and BTNL8, a bona fide TCR-V_γ_ ligand that is similarly expressed at steady state and is stress-labile^15,20^. This normality sensing mode of TCR stimulation may be linked to the unusually high TCR activation threshold acquired during Skint1 selection^50^, which could conceivably limit overt IEL activation in the absence of stress. In contrast to natural killer cells, which sense stress through missing self, DETC Skint1 sensing is a positive regulator of many components of tissue immunosurveillance, a role that may be phenocopied by BTNL sensing by murine and human gut IELs. The clinical implication of our findings is that epithelial pathologies may not always reflect failures to detect infectious, allergic or carcinogenic challenges but rather failings in the maintenance of normal homeostatic interactions before exposure to those challenges occur.

## Methods

### Mice

FVB/NJ strains (wild-type, FVB.Tac^16^, *Trgv5*^–/–^*Trdv4*^–/–^ (ref. ^12^), *Skint1*-transgenic FVB.Tac^24^, huLangerin-DTA^51^, *Tcrd*^–/–^ (ref. ^52^) and FVB.Tac.*Tcrd*^–/–^), C57BL/6J strains (wild-type, Nur77–GFP^36^, *Tcrd*^–/–^ and *Tnfsf9*^–/–^ (ref. ^53^)) and C57BL/6N strains (*Skint1*^–/–^ and *Skint* locus^–/–^)^54^ were bred and maintained under specific pathogen-free conditions in the Biological Research Facility at the Francis Crick Institute with a 12-h light/12-h dark cycle and access to food and water ad libitum at a temperature of 19–23 °C and humidity of 55 ± 10%. Procedures were performed on mice of both sexes aged 6–15 weeks (with the exception of reconstitution experiments below) and were authorized by a UK Home Office License to A.C.H. and the Animal Welfare and Ethical Review Board of The Francis Crick Institute.

### Neonatal DETC reconstitution

FVB *Tcrd*^–/–^ and FVB.Tac *Tcrd*^–/–^ pups at 1–3 d of age were transferred with a thymocyte suspension prepared from E16–E18 FVB wild-type fetuses by intraperitoneal injection. The equivalent of four to five fetal thymi were transferred into each pup. Reconstituted mice were analyzed at 8 weeks of age.

### Antibody administration

All antibodies were administered by a single i.d. injection into the ear pinna using a 30-gauge needle under inhalation anesthesia. For inhibition of Skint1 for confocal imaging, 2 μg of anti-Skint1 (clone 2G2) or rat IgG2a isotype control (BioLegend) were administered in 25 μl of PBS. For all other inhibition experiments, mice were administered 10 μg of anti-Skint1 (BioLegend), anti-4-1BBL (BioXCell), anti-GITRL (BioLegend) or rat IgG2a isotype control (BioLegend) in 10 μl of PBS. For antibody-mediated stimulation of receptors, mice were administered 2 μg of agonistic anti-4-1BB (R&D Systems) and anti-GITR (BioXCell) or respective isotype controls rat IgG2a (BioLegend) and rat IgG2b (BioXCell) in 10 μl of PBS. Anti-Skint1 hybridoma supernatant was produced by the Monoclonal Antibody Core Facility at the Helmholtz Centre Munich, and the antibody was purified by Cell Services at The Francis Crick Institute.

### TEWL measurement

Under inhalation anesthesia, TEWL of the ear pinnae was measured using a Tewameter TM300 probe (Ck Electronic) under constant temperature and humidity conditions. Values were recorded as the average of ten consecutive measurements once readings were stabilized using MPA software (Ck Electronic).

### UVR model

Under inhalation anesthesia, mice were positioned under a cardboard box, and one or both ears were exposed and fixed flat using Blu-Tac. UVB radiation (302 nm) was administered to dorsal ear skin for a dose of 45 mJ cm^–2^ (C57BL/6) or 30 mJ cm^–2^ (FVB) using an XX-15 Series UV bench lamp (UVP) mounted on a Perspex and plastic housing.

### Ex vivo ear skin culture

Ears were split and floated on complete RPMI (10% fetal calf serum, penicillin/streptomycin, 1× non-essential amino acids, 1 mM sodium pyruvate, 10 mM HEPES and 50 μM β-mercaptoethanol) containing 10 μg ml^–1^ anti-Skint1 or isotype control antibodies or 20 μM PP2 (Sigma) or DMSO vehicle alone. After 4 h of culture at 37 °C, ear skin was irradiated with 45 mJ cm^–2^ UVB as described above and returned to 37 °C for 24 h.

### ATP challenge

Mice were injected (i.d.) in the ear with 400 nmol ATP (Sigma) in 10 μl of PBS.

### Irritant contact dermatitis model

Under inhalation anesthesia, 8 μl of 0.5% DNFB (Sigma) in 4:1 acetone:olive oil vehicle was applied to the dorsal ear skin and allowed to dry.

### Flow cytometry

Epidermis was separated from dermis by floating split ear sheets on trypsin-GNK (2.5 mg ml^–1^ trypsin (Sigma-Aldrich), 7.35 mg ml^–1^ NaCl, 0.35 mg ml^–1^ KCl and 0.85 mg ml^–1^ glucose) for 1 h at 37 °C. Single-cell epidermal suspensions were prepared by subsequently digesting epidermal sheets in trypsin-GNK with 1× DNase buffer (1.21 mg ml^–1^ Tris base, 0.5 mg ml^–1^ MgCl_2_ and 73 μg ml^–1^ CaCl_2_) and 100 μg ml^–1^ DNase I (Roche) for 15 min at 37 °C with shaking at 700 r.p.m. Trypsin was neutralized with complete DMEM (Gibco), and the suspension was washed. Cells were stained with Live/Dead Fixable Near-IR dye (Molecular Probes) in PBS for 10 min at room temperature and washed in FACS buffer (PBS, 2% fetal calf serum, 2 mM EDTA and 0.04% sodium azide). Cells were blocked with mouse Fc Block (BD) and stained with combinations of the following conjugated antibodies, which are also listed in Supplementary Table 3, for 20 min at 4 °C: anti-CD3-BV510, anti-CD45-APC, anti-CD45-FITC, anti-CD45-PerCPCy5.5, anti-CD45.1-BV510, anti-CD69-PECy7, anti-CD86-APC, anti-CD86-FITC, anti-I-A/I-E-BV605, anti-TCRδ-BV421, anti-TCRδ-FITC, anti-GITR-APC (all BioLegend), anti-CD69-PE (eBioscience), anti-CD5-BV711 and anti-TCRβ-BV605 (all BD). Cells were then washed before immediate acquisition or alternatively fixed in CellFix (BD) for 10 min at 4 °C. Absolute cell counts were calculated using CountBright beads (Invitrogen). For apoptosis measurement, cells were washed in Annexin-V-binding buffer (BD) following surface staining, incubated with Annexin-V–APC (BioLegend) for 20 min at 4 °C, washed and immediately acquired. For staining of specific γδ TCRs, epidermal cells were rested overnight in complete RPMI at 37 °C before live/dead dye staining and were stained with 17D1 hybridoma supernatant^55^ at 4 °C for 20 min. Cells were then washed and stained with surface antibodies as above, including anti-V_γ_5-APC (BioLegend) and anti-rat IgM-PE (eBioscience). For analysis of other trypsin-sensitive epitopes, the epidermis was separated and digested as above but with TrypLE Express (Gibco) in place of trypsin-GNK, and the epidermis was separated for 2 h. Cells were stained as above but including combinations of the following antibodies: anti-CD122-PE, anti-4-1BB-APC and anti-4-1BB-PE (all BioLegend). Cells were acquired with an LSR II or Fortessa (BD) and analyzed with FlowJo (TreeStar). Gating strategies are detailed in Supplementary Fig. 1.

### Confocal microscopy

For analysis of DETC dendrites and V_γ_5 staining, ears were depilated with Nair and split, and the epidermis was separated by incubation in 0.5 M NH_4_SCN for 30 min at 37 °C and fixed in ice-cold acetone for 20 min at –20 °C.

For analysis of γH2A.X staining and for KC F-actin visualization in Extended Data Fig. 1, whole depilated split ear sheets were flattened in a histology cassette and fixed in neutral buffered formalin for 2 h at room temperature and permeabilized in 0.5% Triton X-100 for at least 1 h at room temperature.

All ear tissue was then washed in PBS with 1% bovine serum albumin (with 0.5% Triton X-100 for whole sheets), blocked with 5% normal goat serum for 1 h at room temperature and stained with combinations of the following antibodies and dyes (see also Supplementary Table 3) overnight at 4 °C: anti-CD3-Pacific Blue, anti-I-A/I-E-AF647, anti-V_γ_5-APC (all BioLegend), anti-V_γ_5-FITC (BD), anti-γH2A.X-FITC (Sigma-Aldrich), AF546-phalloidin or rhodamine-phalloidin (all Molecular Probes). Tissue was then washed and mounted in Vectashield Antifade mounting medium. Microscopy was performed using an LSM 710 (Zeiss; DETC dendrites) or TCS SP5 (Leica; γH2A.X) laser-scanning confocal microscope using a ×40 oil-immersion lens. All images were analyzed in Imaris (Bitplane).

For dendrite analysis, CD3^+^ cells (or V_γ_5^+^ cells in the reconstitution experiments) were identified by the surfaces tool and used to mask F-actin staining. TCR- and F-actin-rich dendrites were identified by using the spots tool to detect 3-μm-diameter volumes containing an MFI in the top 25% of their respective channels. Colocalized CD3 and F-actin spots were selected for quantification on a per cell basis.

For γH2A.X analysis, the cell function was used to identify γ-H2A.X^+^ nuclei by filtering for the top 20% of cells by quality. Nuclei were quantified per image and averaged over three images per mouse.

### iSIM

Epidermal sheets were prepared, separated and fixed in acetone as above and blocked in 5% normal goat serum for 1 h at room temperature. Sheets were stained with anti-Skint1 for 1 h at 37 °C, washed and stained with anti-rat IgG AF568 (Invitrogen), anti-CD3-FITC, anti-V_γ_5-FITC (all BD) and anti-TCRδ-AF647 (BioLegend). iSIM was performed using a VT-iSIM microscope (VisiTech International) with a ×100/1.45-NA objective. Raw images were processed with the Richardson–Lucy deconvolution algorithm and analyzed with NIS-Elements (Nikon) or Fiji (NIH) software. Skint1 staining relative to T cell bodies was quantified using Definiens Developer XD software (Definiens). Individual cells and Skint1 particles were detected using Otsu’s automatic multilevel thresholding and multiresolution segmentation algorithms. The MFI of Skint1 particles within and outside of T cell body masks was quantified.

### Histology

Whole ears were flattened in a histology cassette and fixed in neutral buffered formalin for 24 h and dehydrated in 70% ethanol for 24 h. Ears were embedded, sectioned and stained with H&E by the Experimental Histopathology Facility at The Francis Crick Institute. Sections were photographed using a SlideScanner (Zeiss) at 10× magnification. Epidermal thickness was determined as the average of 30 manual measurements using QuPath software.

### Ear swelling measurement

Total ear thickness was calculated as the average of three measurements of the ear pinna with a micrometer (Mitutoyo) after discarding the first measurement.

### Measurement of CPDs

CPDs were measured by ELISA. Epidermal sheets were separated by NH_4_SCN as above. Epidermal DNA was isolated using a DNeasy Blood and Tissue kit (Qiagen) and denatured at 100 °C for 10 min. DNA (10 ng) was dried onto protamine sulfate-coated 96-well plates (Nunc), and ELISAs were performed using anti-CPDs (Cosmo Bio), biotinylated goat anti-mouse IgG (Invitrogen), streptavidin–horseradish peroxidase (R&D) and a TMB Substrate Reagent set (BD), as per the anti-CPDs manufacturer’s protocol. Absorbance was measured using an Infinite F200 Pro spectrophotometer (Tecan).

### Toluidine blue permeability

Whole ears were dehydrated and rehydrated by sequential 2-min incubations in ice-cold 25%, 50%, 75%, 100%, 75%, 50% and 25% methanol in PBS and incubated in cold PBS for 5 min. Ears were stained in 0.1% (wt/vol) toluidine blue (Sigma-Aldrich) for 2 min and then destained in PBS for 1 min. Ears were photographed with an Stemi SV 11 stereomicroscope (Zeiss). Toluidine blue staining was quantified in Fiji by color deconvolution (H&E setting) and thresholding to measure total ear area (green channel) and toluidine blue area (indigo channel).

### qPCR

For analysis of total epidermal gene expression, epidermal sheets were separated by NH_4_SCN as above and homogenized in buffer RLT (Qiagen) using stainless steel beads (Qiagen) and a TissueLyser II (Qiagen). RNA was isolated using the RNeasy Mini kit (Qiagen) and an RNase-free DNase set (Qiagen).

For analysis of gene expression from DETCs, LCs and KCs, single-cell epidermal suspensions were prepared from ear sheets using trypsin-GNK and stained as above with Live/Dead Fixable Near-IR dye (Molecular Probes) and combinations of anti-CD3-BV510, anti-CD45-APC, anti-CD45-FITC, anti-CD45-Pacific Blue, anti-I-A/I-E-BV605, anti-I-A/I-E-FITC, anti-TCRδ-APC, anti-TCRδ-BV421 (all BioLegend) or anti-TCRδ-PerCPeFluor710 (eBioscience). CD45^+^TCRδ^+^ DETCs, CD45^+^MHCII^+^ LCs and CD45^–^ KCs were sorted directly into buffer RLT Plus (Qiagen) using an Aria Fusion (BD). RNA was isolated using an RNeasy Micro Plus kit (Qiagen).

For analysis of gene expression from 4-1BB^hi^ and 4-1BB^lo^ DETCs, mice were shaved, and epidermal suspensions from ears and trunk skin were prepared using TrypLE Express (Gibco) as described above but with a 4-h incubation to separate the epidermis. Cells were stained with Live/Dead Fixable Near-IR dye (Molecular Probes) and anti-CD45-FITC, anti-TCRδ-BV421 and anti-4-1BB-PE (all BioLegend) and sorted. RNA was isolated as described above.

cDNA was synthesized using Superscript II Reverse Transcriptase, dNTP set and Anchored Oligo(dT)20 primer (Invitrogen). qPCRs were run with PowerUp SYBR Green (Applied Biosystems) and 0.4 μM primer sets listed in Supplementary Table 5 on a ViiA 7 Real-Time PCR System (Thermo Fisher). Threshold cycle (*C*_t_) values were processed using QuantStudio software, and Δ*C*_t_ values were calculated as 2^(reference – gene)^, where the reference was *CypA* (cyclophilin).

### Single-cell RNA sequencing

Wil-type FVB mice were treated with 10 μg of anti-Skint1 or rat IgG2a isotype control by i.d. injection into the ear pinna, as described above. Seven days later, epidermal suspensions were made from ear skin and were stained as described above using Live/Dead Fixable Near-IR dye (Molecular Probes), anti-EpCAM-PE (eBioscience) and anti-CD45-APC and anti-I-A/I-E-FITC (all BioLegend), and live CD45^+^MHCII^–^EpCAM^–^ lymphocytes were sorted using an Aria Fusion (BD). Samples were prepared using the Chromium Single Cell 3′ GEM, Library and Gel Bead kit v3 (10x Genomics). Cell viability was assessed using an EVE cell counter and trypan blue viability stain. Approximately 10,000 cells were loaded into the 10x Chromium, which was operated according to manufacturer’s instructions. Sequencing was performed on an Illumina HiSeq 4000 with a read configuration recommended by 10x Genomics for the sequencing of these libraries (28-base pair (bp) read 1, 98-bp read 2, 8-bp index 1).

### Bioinformatic analysis of single-cell RNA sequencing

Data were processed using CellRanger v3.0.2 using the prebuilt mm10 index v3.0.0, and individual Seurat objects were created. All of the following functions belong to the Seurat v3.1.1 package^56^, and their parameters are mentioned only if differing from the default. The following steps were applied to the data:

1. Cells with less than 500 features, more than 2,500 features and more than 12% mitochondrial reads were excluded.
2. Feature counts were normalized per cell using the NormalizeData function.
3. The FindVariableFeatures function was used to select the 2,000 most variable genes.
4. These genes were used to identify integration anchors (FindIntegrationAnchors, dims = 1:20, anchor.features = 2050), and the two samples (isotype control and anti-Skint1) were integrated using the IntegrateData function.
5. The expression of each gene was centered using the ScaleData function parametrized with all the available features.
6. Principal component analysis was performed with the RunPCA function (npcs = 100).
7. Clustering was performed using the FindNeighbors function (dims = 1:40) and the FindClusters function (resolution = 1.18).
8. Dimension reduction was performed using RunUMAP (dims = 1:40).
9. Clusters were manually annotated using the following marker genes: *Ptprc*, *Cd3d*, *Cd3e*, *Trdc*, *Trgv5*, *Trdv4*, *Il17a*, *Scart2*, *Trac*, *Cd8a*, *Cd8b1*, *Cd4*, *Foxp3*, *Il10*, *Ncr1*, *Rora*, *Il7r*, *Csf3r*, *Cd207*, *Itgam*, *Krt5* and *Krt10*.
10. DETCs were identified by the expression of *Ptprc*, *Cd3d*, *Cd3e*, *Trdc*, *Trgv5* and *Trdv4*, and their cell IDs were extracted.

The original Seurat objects were filtered using DETC IDs to remove other cell types from the analysis. Steps 1 to 8 were reapplied to the resulting data with the following differences: FindNeighbors (dims = 1:23), FindClusters (resolution = 0.28) and RunUMAP (dims = 1:23).

Marker genes for each cluster were identified using the FindAllMarkers function (only.pos = F, logfc.threshold = 0, min.pct = 0, min.cells.feature = 0 and min.cells.group = 0 and return.thresh = 2). For each cluster, marker genes were sorted by avg_logFC (increasing order), and GSEA v3.0 (ref. ^57^) was run for the C5 v6.2 ontology gene sets with the following parameters: -nperm 1000, -scoring_scheme classic and -set_min 3 and -set_max 50000. Likewise, for each cluster, marker genes were filtered (avg_logFC>0, p_val_adj<0.05) and used to perform a gene ontology analysis using the enrichGO function of the clusterProfiler v3.12.0 package^58^ (pAdjustMethod = BH, pvalueCutoff = 0.01 and qvalueCutoff = 0.05) with the org.Mm.eg.db v3.8.2 annotation.

Differential expression analysis between the two experimental conditions was performed using the FindMarkers function of Seurat (logfc.threshold = 0, min.pct = 0, min.cells.feature = 0 and min.cells.group = 0). The resulting gene list was sorted by avg_logFC (decreasing order), and GSEA v3.0 was run for the Skint1 selection signature with the following parameters: -nperm 1000, -scoring_scheme classic, -set_min 3 and -set_max 50000. The Skint1 selection signature was generated from the top 200 genes overexpressed in E15 V_γ_5^+^ thymocytes from wild-type FVB versus Tac embryos (Supplementary Table 1), as determined by Illumina microarray analysis and described previously^35^.

Expression plots and violin plots were produced using the normalized counts.

### Bulk RNA sequencing

Wild-type C57BL/6 mice were irradiated with 45 mJ cm^–2^ UVB as described above or were left unchallenged. Dorsal ear skin was collected at 24 h after UVR, and single-cell suspensions were made and stained as described above with Live/Dead Fixable Near-IR dye (Molecular Probes) and anti-CD3-PerCPCy5.5, anti-CD45-APC, anti-I-A/I-E-BV605 and anti-TCRδ-BV421 (all BioLegend). Live CD45^+^CD3^+^TCRδ^+^ DETCs were sorted directly into buffer RLT Plus (Qiagen) using an Aria Fusion (BD), and RNA was isolated using an RNeasy Micro Plus kit (Qiagen). cDNA was prepared from 10 ng of input RNA using the Ovation RNA-Seq System V2 (NuGEN). Final libraries were then constructed using the Ultralow Library System V2 (NuGEN). The resulting libraries were pooled for sequencing on an Illumina HiSeq 2500 platform with single-ended 75-bp reads.

### Bioinformatic analysis of bulk RNA sequencing

Raw reads were quality and adapter trimmed using cutadapt v1.9.1 (ref. ^59^) before alignment. Reads were then aligned and quantified using RSEM v1.3.0 and STAR v2.5.2 (refs. ^60,61^) against the mouse genome GRCm38 and annotation release 86, both from Ensembl. Differential gene expression analysis was performed in R using the DESeq2 v1.24.0 package^62^. Differential genes were selected using a 0.05 false discovery rate threshold. Normalization and VST was applied on raw counts before performing principal component analysis and Euclidean distance-based clustering. For the heat maps, *Z* scores were computed on the VST of the raw counts. Heat maps were made in R using the ComplexHeatmap v2.0.0 package^63^. GSEA was run as described above using gene signatures created from the top 200 overexpressed genes in each DETC cluster from the single-cell RNA-sequencing experiment.

### Statistics

Data were analyzed in Prism 9.3.0 (GraphPad) and R v3.6.0. Statistical tests were performed as indicated in the figure legends, and *n* values are also provided. All error bars represent mean ± s.d. Unless explicitly stated in the figure legends, all dots and *n* values refer to individual mice. Mice were randomly assigned to treatment groups where applicable. No data were excluded. Data were presumed to be normally distributed, and all tests were two tailed where applicable. Statistical significance was defined as *P* < 0.05, and all significant *P* values are shown in the figures. Welch’s correction was applied to *t*-tests where variance was significantly different between sample groups as determined by an *F*-test.

### Reporting Summary

Further information on research design is available in the Nature Research Reporting Summary linked to this article.

## Online content

Any methods, additional references, Nature Research reporting summaries, source data, extended data, supplementary information, acknowledgements, peer review information; details of author contributions and competing interests; and statements of data and code availability are available at 10.1038/s41590-021-01124-8.

## Supplementary information

Supplementary Information Supplementary Fig. 1 and Tables 1–5.

Reporting Summary

## Data Availability

RNA-sequencing datasets generated in this paper are available under accession codes GSE160476 (single cell) and GSE160477 (bulk). Previously published datasets used in this paper are provided in the Supplementary Tables. The public gene set used in Extended Data Fig. 3e is available at GO:0050778. Source data are provided with this paper.

## Source data

Source Data Fig. 1 Statistical source data.
Source Data Fig. 2 Statistical source data.
Source Data Fig. 3 Statistical source data.
Source Data Fig. 4 Statistical source data.
Source Data Fig. 5 Statistical source data.
Source Data Fig. 6 Statistical source data.
Source Data Fig. 7 Statistical source data.
Source Data Extended Data Fig. 1 Statistical source data.
Source Data Extended Data Fig. 2 Statistical source data.
Source Data Extended Data Fig. 3 Statistical source data.
Source Data Extended Data Fig. 4 Statistical source data.
Source Data Extended Data Fig. 5 Statistical source data.
Source Data Extended Data Fig. 6 Statistical source data.

## Code availability

Code used for single-cell RNA-sequencing processing is available at https://github.com/bahnk/scRNASeq-DETCs.
