# Supplementary material for: Normality sensing licenses local T cells for innate-like tissue surveillance
Source: Nat Immunol. 2022 Feb 14;23(3):411–22. doi: 10.1038/s41590-021-01124-8 (PMC8901436; doi:10.1038/s41590-021-01124-8)
Supplement: Supplementary file 1 — Supplementary Fig. 1 and Tables 1–5. [file 41590_2021_1124_MOESM1_ESM.pdf]

nature portfolio

https://doi.org/10.1038/s41590-021-01124-8

Supplementary information

Normality sensing licenses local T cells for innate-like tissue surveillance

In the format provided by the authors and unedited

Supplementary Information

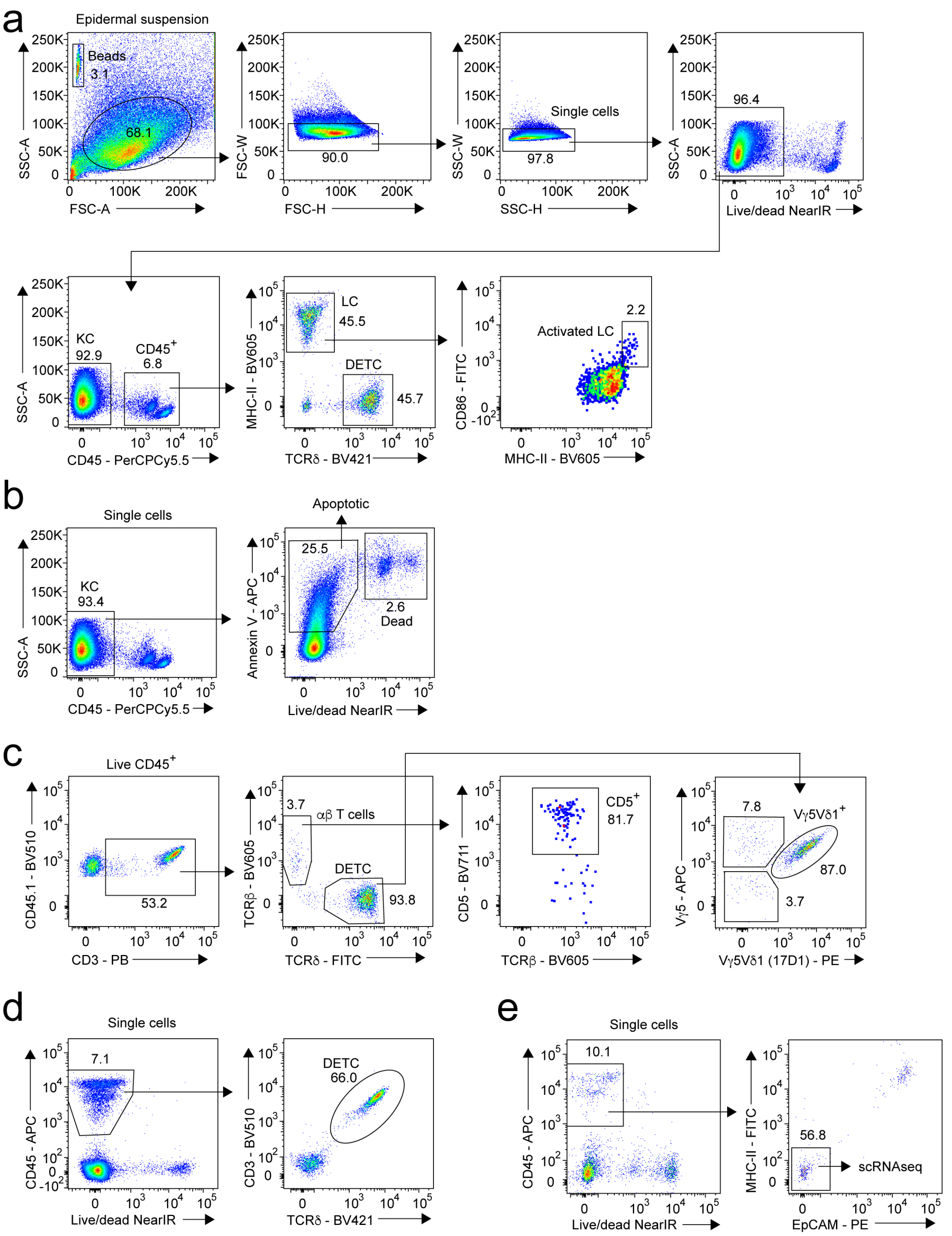

Supplementary Figure 1: Flow cytometry gating strategies. a) Gating of epidermal DETCs, LCs and KCs for quantification, phenotyping and sorting. **b**) Gating of KC for apoptosis analysis. **c**) Gating of epidermal T cells for TCR phenotyping. **d**) Gating for sorting or routine phenotyping of DETCs. **e**) Negative gating for sorting of epidermal T cells for scRNAseq. Exact fluorophores used were changed between individual experiments depending upon downstream phenotyping.

Supplementary Table 1: Thymic Skint1 selection gene signature in DETCs.

Top 200 genes upregulated in wild-type vs FVB.Tac Vγ5Vδ1^{+} progenitors from E15-16 embryonic thymus as measured by Illumina microarray analysis, as published in Turchinovich & Hayday 2011 (ref. 35).

ENO3	EIF5	UBE2K	LOC100042405	LSM3
CTSW	EIF4A2	PFKP	A230050P20RIK	LOC383712
LOC100047353	HNRNPH1	CCND2	ACAA2	RPS15
XCL1	LOC626309	UBLCP1	GTPBP4	SCL0001978.1_6
EGR3	BC003885	GNA-RS1	LOC433955	RASA3
ENSMUSG00000068790	RNF19A	SDCBP	WDR75	RPS27A
PPIC	CISH	2810026P18RIK	SH2D2A	PPAN
EPHX1	ADD3	SEPT12	IGFBP7	PTPN6
HSPA1A	NASP	A930008A22RIK	2810407C02RIK	CENTD3
SCOTIN	CD28	GTF2F2	LOC623453	AP1M1
RBBP7	IRF2	LOC100047651	DNAJB1	PGK1
GADD45G	LOC381738	AK2	ITM2A	NUP205
HNRNPA2B1	TPM4	SUMO1	PPIF	2610101N10RIK
ENDOD1	E330016A19RIK	9430008C03RIK	NIP7	HIST2H2BE
TAGAP	LGALS1	IGF2R	ACTB	IGF2
LOC100048845	DDIT4	RABGGTB	1190002H23RIK	SEPT16
VIM	HSPA8	SLC38A2	TPI1	HMGB2L1
KLF2	2610029G23RIK	LOC433749	BIRC5	1110012O05RIK
LTB	HSP105	DARS	CSNK2A2	GSPT1
PRKCH	SNORA65	UMPS	LOC100041569	UCK2
RGS16	ACADM	SUMO3	D030056L22RIK	ZFP330
CHCHD10	MAPRE1	PLP2	PGLYRP1	EDF1
3300001P08RIK	PGP	SFXN1	MLF2	SCL0002315.1_12
MYO1G	ZFP36L1	NDFIP1	2310008M10RIK	AKR1C12
NFKBID	EIF4G2	PPP2CA	LMNA	LCP1
KLHDC2	ANXA2	GLIPR2	HDGF	MTDNA_ND2
HSPD1	RWDD1	PRICKLE3	HIP2	SEC11A
NT5E	NACA	A130082M07RIK	GNL3	CYP51
BTG1	MSN	ADSS	LOC100048037	EZH2
ZYX	LOC329750	ZRANB2	HIST1H4A	PPTC7
CYTIP	EMP3	MORF4L1	LOC381398	MOBK1B
PKP4	LOC331102	PDLIM1	AKR1C18	VDAC3
CD160	HSPA1B	UCHL5	MCM7	GEMIN6
PRR13	H19	NFKBIA	D19BWG1357E	COQ5
AI850995	SMN1	HSF1	EIF5A	ARPC5
EG434858	ISY1	MED14	CD9	0610007J10RIK
TPM3	MYL6	ELK3	CD69	HIST1H3D
NRGN	IVNS1ABP	SLC35C1	LOC100047834	PRRC1
LOC671878	SLC29A1	SHMT1	ZNFN1A3	MKI67IP
CLK1	LOC100045617	ZDHHC6	EHD4	NFYB

Supplementary Table 2: Induction of TNF superfamily receptors by Skint1 selection of DETCs.

Expression of TNF superfamily receptors in wild-type vs FVB.Tac $V\gamma5V\delta1^+$ progenitors from E15-16 embryonic thymus. Gene expression was measured by Illumina microarray analysis, as published in Turchinovich & Hayday 2011 (ref. 35).

Gene	Mean signal wild-type	Mean signal FVB.Tac	Fold change wild-type: FVB.Tac
Tnfrsf9	334.62615	88.57383	3.77793475
Tnfrsf18	453.9273	121.3864	3.73952354
Tnfrsf4	204.0369	98.90794	2.06289707
Tnfrsf13b	237.4174	119.32379	1.9896904
Tnfrsf22	198.17855	103.077795	1.92261146
Tnfrsf1b	141.3792	93.73878	1.5082253
Tnfrsf12a	145.4694	102.3394	1.42144081
Tnfrsf13c	105.0007	76.09886	1.37979334
Tnfrsf19	135.2836	111.1403	1.21723263
Tnfrsf21	166.61825	138.140765	1.20614831
Tnfrsf1a	109.2932	95.17151	1.14838149
Tnfrsf25	159.9612	144.9653	1.10344476
Tnfrsf10b	157.8602	177.2649	0.89053276

Supplementary Table 3: Antibodies and similar reagents used in flow cytometry, imaging and ELISA.

Reagent	Source	Identifier	Dilution
anti-CD16/CD32 Fc block	BD	Cat#553141	1:200
anti-CD3 BV510 (17A2)	BioLegend	Cat#100234	1:300
anti-CD3 FITC (2C11)	BD	Cat#553061	1:300
anti-CD3 Pacific Blue (17A2)	BioLegend	Cat#100214	1:200
anti-CD3 PE (2C11)	BioLegend	Cat#100308	1:300
anti-CD3 PerCPCy5.5 (17A2)	BioLegend	Cat#100218	1:300
anti-CD5 BV711 (53-7.3)	BD	Cat# 740649	1:300
anti-CPDs (TDM-2)	Cosmo Bio	Cat#CAC-NM-DND-001	1:1000
anti-CD45 APC (30-F11)	BioLegend	Cat#103112	1:300
anti-CD45 FITC (30-F11)	BioLegend	Cat#103108	1:300
anti-CD45 Pacific Blue (30-F11)	BioLegend	Cat#103126	1:300
anti-CD45 PerCPCy5.5 (30-F11)	BioLegend	Cat#103132	1:300
anti-CD45.1 BV510 (A20)	BioLegend	Cat#110741	1:300
anti-CD69 PE (H1.2F3)	eBioscience	Cat#12-0691-83	1:300
anti-CD69 PECy7 (H1.2F3)	BioLegend	Cat#104512	1:300
anti-CD86 APC (GL-1)	BioLegend	Cat#105012	1:300
anti-CD86 FITC (GL-1)	BioLegend	Cat# 105006	1:300
anti-CD122 PE (TM-β1)	BioLegend	Cat#123210	1:300
anti-CD137 (4-1BB) (158321)	R&D Systems	Cat#MAB9371-500	0.2mg/ml (in vivo)
anti-CD137 (4-1BB) APC (17B5)	BioLegend	Cat#106110	1:300
anti-CD137 (4-1BB) PE (17B5)	BioLegend	Cat#106106	1:300
InVivoMab anti-CD137L (4-1BBL) (TKS-1)	BioXCell	Cat#BE0110-1MG	1mg/ml (in vivo)
InVivoMab anti-GITR (DTA-1)	BioXCell	Cat#BE0063-1MG	0.2mg/ml (in vivo)
anti-EpCAM (CD326) PE (G8.8)	eBioscience	Cat#12-5791-82	1:300
anti-GITR APC (DTA-1)	BioLegend	Cat#126311	1:300
LEAF purified anti-GITRL (5F1)	BioLegend	Cat#147404	1mg/ml (in vivo)
anti-I-A/I-E (MHC-II) AF647 (M5/114.15.2)	BioLegend	Cat#107618	1:500
anti-I-A/I-E (MHC-II) BV605 (M5/114.15.2)	BioLegend	Cat#107639	1:300
anti-I-A/I-E (MHC-II) FITC (M5/114.15.2)	BioLegend	Cat#107606	1:300
anti-IgM PE (HIS40)	eBioscience	Cat#12-0990-82	1:200
anti-mouse IgG biotin (polyclonal)	Invitrogen	Cat#62-6540	1:1000
anti-rat IgG AF568 (polyclonal)	Invitrogen	Cat#A-11077	1:400
anti-Skint1 (2G2)	Helmholtz Monoclonal	Salim et al 2016 (ref. 19)	1:100 (iSIM)

	Antibody Facility		1mg/ml (in vivo)
anti-TCRβ BV605 (H57-597)	BD	Cat# 562840	1:300
anti-TCRδ (GL3) AF647	BioLegend	Cat#118134	1:300
anti-TCRδ (GL3) APC	BioLegend	Cat#118116	1:300
anti-TCRδ (GL3) BV421	BioLegend	Cat#118119	1:300
anti-TCRδ (GL3) FITC	BioLegend	Cat#118106	1:300
anti-TCRδ (GL3) PerCPeFluor710	eBioscience	Cat#46-5711-82	1:300
anti-Vγ5 (536) APC	BioLegend	Cat#137506	1:300 (FC) 1:100 (IF)
anti-Vγ5 (536) FITC	BD	Cat#553229	1:300
anti-Vγ5Vδ1 (17D1)	R. Tigelaar, J. Lewis	Mallick-Wood et al. 1998 (ref. 55)	Neat (S/N)
anti-γH2A.X FITC (JBW301)	Sigma-Aldrich	Cat#16-202A	1:100
Ultra-LEAF purified rat IgG2a isotype control (RTK2758)	BioLegend	Cat#400565	0.2-1mg/ml (in vivo)
InVivoMab rat IgG2b isotype control (LTF-2)	BioXCell	Cat#BE0090-1MG	0.2mg/ml (in vivo)
**Non-antibody staining reagents**			
LIVE/DEAD Fixable Near-IR Dye	Molecular Probes	Cat#L10119	1:1000
Annexin V APC	BioLegend	Cat#640920	1:25
Alexa Fluor 546 Phalloidin	Molecular Probes	Cat#A22283	1:200
Rhodamine Phalloidin	Molecular Probes	Cat#R415	1:50
CellFIX	BD	Cat#340181	1:10
CountBright beads	Invitrogen	Cat#C36950	5000/sample
Annexin V Binding Buffer	BioLegend	Cat#422201	Neat
Streptavidin-HRP	R&D Systems	Cat#890803	1:200
Toluidine Blue	Sigma-Aldrich	Cat# 89640-5G	0.1% (w/v)

Supplementary Table 4: Commercial kits and reagents used in tissue processing and nucleic acid analysis.

Reagent	Source	Identifier
TMB Substrate Reagent set	BD	Cat#555214
DNeasy Blood and Tissue kit	Qiagen	Cat#69504
RNeasy Mini kit	Qiagen	Cat#74106
RNeasy Micro Plus kit	Qiagen	Cat#74034
RNase-free DNase set	Qiagen	Cat#79254
Superscript II Reverse Transcriptase	Invitrogen	Cat#18064014
dNTP Set	Invitrogen	Cat#10297018
Anchored Oligo(dT)20 Primer	Invitrogen	Cat#12577011
PowerUp SYBR Green Master Mix	Applied Biosystems	Cat#A25777
Trypsin from bovine pancreas	Sigma-Aldrich	Cat#T1005-5G
TrypLE Express	Gibco	Cat#12604013
DNase 1 from bovine pancreas	Roche	Cat#11284932001
DMEM, high glucose	Gibco	Cat#41965062
RPMI 1640	Gibco	Cat#21875091
Chromium Single Cell 3’ GEM, Library & Gel Bead Kit v3	10x Genomics	Cat#PN-1000075
Ovation RNA-Seq System V2	NuGEN	Cat#7102-32
Ultralow Library System V2	NuGEN	Cat#0344NB-32

Supplementary Table 5: Primer sets used for quantitative PCR.

Target	F primer (5’→3’)	R primer (5’→3’)
Cxcl2	TGAACAAAGGCAAGGCTAACT G	CAGGTACGATCCAGGCTTCC
CypA	CAAATGCTGGACCAAACACAA	CCATCCAGCCATTCAGTCTTG
Egr1	CCTATGAGCACCTGACCACA	TCGTTTGGCTGGGATAACTC
Gzmb	ACAAAGGCAGGGGAGATCAT	CGAATAAGGAAGCCCCCACA
Gzmc	CCCGTCCCTACATGGCATATT	GCTTTTGCCACAGGGATGAT
Gzmf	CAGGGCATGTTTGCAGTGTG	CCGGAGTCACCACTGTAAGTAGA
Havcr2	AGACATCAAAGCAGCCAAGGT	TCCGTGGTTAGGGTTCTTGG
Il2rb	TAAAGACCTGCGACTTCCATC	AGTGAGAGACCTGGGAGAC
Il13	GCCAGGTCCACACTCCATA	GCTTATTGAGGAGCTGAGCAA
Krt1	CTAAGTTTTGGGTCCGGGTT	AGTTTGCCTCCTTCATCGAC
Krt5	CCTGCAGAAGGCCAAGCA	TGGTGTTCATGAGCTCCTGGT
Krt10	GCCTCCTACATGGACAAAGTC	GCTTCTCGTACCACTCCTTGA
Krt14	GCAGTATCCGATCTCTTCATGC	GGCCCTCGAATCCTCTGACT
Lor	GAGGTCTTTCCACAACCCAC	TCCCTCACTCATCTTCCCTG
Ptpn6	TGGCCATGCATCAGCCTTGA	GTCCTAGGGTCTGGTGAGGG
Rps27	CCTCCCTACGAGAACATGCC	GACTACCGTTTGTGCATGGC
Rps29	TTCCTTTCTCCTCGTTGGGC	TTCAGCCCGTATTTGCGGAT
Skint1	AAACAAAAGGGAGCTGACCC	CCCCTCTAAGCCGTTCACTA
Skint2	GCTACAGGAGTACTTCTCTGTG TTGT	TGGTGCCAAGACTGGCCT
Tgm1	CCTTGAGCTCCTCATTGGAA	CCCTTACCCACTGGGATGAT
Tnfrsf9	CGTGCAGAACTCCTGTGATAAC	GTCCACCTATGCTGGAGAAGG
Tnfrsf18	AAGGTTCAGAACGGAAGTG	GGGTCTCCACAGTGGTACT
Tigit	TGAAGGTCCAAGAAAGCTCAG	TAAGCAAATGAGTCCCAGCA
Xcl1	GACTTCTCCTCCTGACTTTCCT G	GGACTTCAGTCCCCACACC
