# Supplementary material for: Normality sensing licenses local T cells for innate-like tissue surveillance
Source: Nat Immunol. 2022 Feb 14;23(3):411–22. doi: 10.1038/s41590-021-01124-8 (PMC8901436; doi:10.1038/s41590-021-01124-8)
Supplement: Supplementary file 2 — Reporting Summary [file 41590_2021_1124_MOESM2_ESM.pdf]

nature portfolio

Corresponding author(s): Adrian Hayday
Last updated by author(s): Dec 9, 2021

Reporting Summary

Nature Portfolio wishes to improve the reproducibility of the work that we publish. This form provides structure for consistency and transparency in reporting. For further information on Nature Portfolio policies, see our Editorial Policies and the Editorial Policy Checklist.

Statistics

For all statistical analyses, confirm that the following items are present in the figure legend, table legend, main text, or Methods section.

n/a	Confirmed	
☐	☒	The exact sample size (*n*) for each experimental group/condition, given as a discrete number and unit of measurement
☐	☒	A statement on whether measurements were taken from distinct samples or whether the same sample was measured repeatedly
☐	☒	The statistical test(s) used AND whether they are one- or two-sided<br>*Only common tests should be described solely by name; describe more complex techniques in the Methods section.*
☐	☒	A description of all covariates tested
☐	☒	A description of any assumptions or corrections, such as tests of normality and adjustment for multiple comparisons
☐	☒	A full description of the statistical parameters including central tendency (e.g. means) or other basic estimates (e.g. regression coefficient) AND variation (e.g. standard deviation) or associated estimates of uncertainty (e.g. confidence intervals)
☐	☒	For null hypothesis testing, the test statistic (e.g. *F*, *t*, *r*) with confidence intervals, effect sizes, degrees of freedom and *P* value noted<br>*Give P values as exact values whenever suitable.*
☒	☐	For Bayesian analysis, information on the choice of priors and Markov chain Monte Carlo settings
☒	☐	For hierarchical and complex designs, identification of the appropriate level for tests and full reporting of outcomes
☒	☐	Estimates of effect sizes (e.g. Cohen's *d*, Pearson's *r*), indicating how they were calculated

Our web collection on statistics for biologists contains articles on many of the points above.

Software and code

Policy information about availability of computer code

Data collection	Data were collected with FACSDiva v8.0.1 (BD), Zen 2010 (Zeiss), Leica Application Suite Advanced Fluorescence (Leica), i-control v3.7.3 (Tecan) and QuantStudio v1.2 (Thermo Fisher).
Data analysis	Data were analyzed in FlowJo v10.3 (Treestar), Imaris v 9.5.1 (Bitplane), NIS Elements (Nikon), Definiens Developer XD v2.7 (Definiens), ImageJ v1.52t (NIH), QuPath v0.1.2, Prism 9.3.0 (GraphPad), and R v3.6.0. including packages CellRanger v.3.0.2, Seurat v3.1.1, cutadapt v1.9.1, RSEM v.1.3.0, STAR v2.5.2, DESeq2 v1.24.0, ComplexHeatmap v2.0.0.

For manuscripts utilizing custom algorithms or software that are central to the research but not yet described in published literature, software must be made available to editors and reviewers. We strongly encourage code deposition in a community repository (e.g. GitHub). See the Nature Portfolio guidelines for submitting code & software for further information.

Data

Policy information about availability of data

All manuscripts must include a data availability statement. This statement should provide the following information, where applicable:

- Accession codes, unique identifiers, or web links for publicly available datasets
- A description of any restrictions on data availability
- For clinical datasets or third party data, please ensure that the statement adheres to our policy

RNA sequencing datasets generated in this paper are available under accession codes GSE160476 (single cell) and GSE160477 (bulk). All source data are provided with this paper. Previously published datasets used in this paper are provided in supplementary tables. Public gene set used in Extended data Fig 3e is available at GO:0050778.

Field-specific reporting

Please select the one below that is the best fit for your research. If you are not sure, read the appropriate sections before making your selection.

☒ Life sciences ☐ Behavioural & social sciences ☐ Ecological, evolutionary & environmental sciences

For a reference copy of the document with all sections, see nature.com/documents/nr-reporting-summary-flat.pdf

Life sciences study design

All studies must disclose on these points even when the disclosure is negative.

Sample size	No sample size calculations were performed. Sample sizes were based upon reasonable cohorts of animals, ideally n=5/group per experiment. In all possible cases, repeat experimental data were pooled together to ensure maximum sample sizes per experiment. During repeat experiments or following pilot experiments, sample sizes were adjusted based upon variability of parameters.
Data exclusions	No data were excluded
Replication	All key experiments were repeated at least once as stated in figure legends. Single cell and bulk RNAseq results were validated by independent experiments to the extent possible.
Randomization	Animals were randomly assigned to treatment groups based on housing.
Blinding	Blinding was not performed as no subjective scoring methods were used. All visual data (e.g. imaging, tissue staining, histology) was processed objectively by automated quantification methods to ensure accurate and unbiased reporting.

Reporting for specific materials, systems and methods

We require information from authors about some types of materials, experimental systems and methods used in many studies. Here, indicate whether each material, system or method listed is relevant to your study. If you are not sure if a list item applies to your research, read the appropriate section before selecting a response.

Materials & experimental systems

n/a	Involved in the study	
☐	☒	Antibodies
☒	☐	Eukaryotic cell lines
☒	☐	Palaeontology and archaeology
☐	☒	Animals and other organisms
☒	☐	Human research participants
☒	☐	Clinical data
☒	☐	Dual use research of concern

Methods

n/a	Involved in the study	
☒	☐	ChIP-seq
☐	☒	Flow cytometry
☒	☐	MRI-based neuroimaging

Antibodies

Antibodies used	All antibodies used are listed in Supplementary Table 3.
Validation	Skint1 2G2 was validated in Salim, M. et al. Characterization of a Putative Receptor Binding Surface on Skint-1, a Critical Determinant of Dendritic Epidermal T Cell Selection. J. Biol. Chem. 291, 9310–9321 (2016)<br><br>Commercial antibodies were validated by vendors. Supporting validation statements can be viewed on the manufacturers' website pages for each product with reference to the catalogue numbers as reported in Supplementary Table 3.

Animals and other organisms

Policy information about studies involving animals; ARRIVE guidelines recommended for reporting animal research

Laboratory animals	Mice of both sexes were used at ages 6-15 weeks (except in the case of neonatal reconstitution experiments as described in methods). C57Bl/6J strains used were: wildtype, Nur77-GFP, Tcrd-/- and Tnfsf9-/- . C57Bl/6N strains used were Skint-/- , Skint locus-/- and littermate controls. FVBN/J strains used were wildtype, Tac (carrying Skint1 mutation), Tac.Skint1tg, Vg5-/-Vd1-/-, Tcrd-/- Tac.Skint1tg, Tac.Tcrd-/-, huLangerin-DTA.

	Mice were maintained at The Francis Crick Institute's Biological resource facilities with a 12h light/dark cycle and access to food and water ad libitum, temperature 19–23°C, 55±10% humidity.
Wild animals	Wild animals were not used in this study.
Field-collected samples	No field-collected samples were used in this study.
Ethics oversight	Experiments were authorized by a UK Home Office License to Adrian Hayday and the Animal Welfare and Ethical Review Board of The Francis Crick Institute.

Note that full information on the approval of the study protocol must also be provided in the manuscript.

Flow Cytometry

Plots

Confirm that:

☒ The axis labels state the marker and fluorochrome used (e.g. CD4-FITC).

☒ The axis scales are clearly visible. Include numbers along axes only for bottom left plot of group (a 'group' is an analysis of identical markers).

☒ All plots are contour plots with outliers or pseudocolor plots.

☒ A numerical value for number of cells or percentage (with statistics) is provided.

Methodology

Sample preparation	Epidermis was separated from dermis by floating split ear sheets on trypsin-GNK (2.5mg/ml trypsin (Sigma-Aldrich), 7.35mg/ml NaCl, 0.35mg/ml KCl, 0.85mg/ml glucose) for one hour at 37°C. Single cell epidermal suspensions were prepared by subsequently digesting epidermal sheets in trypsin-GNK with 1X DNase buffer (1.21 mg/ml Tris base, 0.5mg/ml MgCl2, 73µg/ml CaCl2) and 100µg/ml DNase I (Roche) for 15 min at 37°C with 700rpm shaking. Trypsin was neutralized with complete DMEM (Gibco) and suspension was washed. Cells were stained with Live/dead Fixable Near-IR Dye (Molecular Probes) in PBS for 10 min at RT, then washed in FACS buffer (PBS 2% FCS 2mM EDTA 0.04% sodium azide). Cells were blocked with mouse Fc Block (BD) and stained with combinations of the following conjugated antibodies, also listed in Table S3, for 20 min at 4°C: anti-CD3-BV510, anti-CD45-APC, anti-CD45-FITC, anti-CD45-PerCPCy5.5, anti-CD45.1-BV510, anti-CD69-PECy7, anti-I-A/I-E-BV605, anti-TCRδ-BV421, anti-TCRδ-FITC, anti-GITR-APC (all BioLegend), anti-CD69-PE (eBioscience), anti-CD5-BV711 and anti-TCRβ-BV605 (all BD). Cells were then washed before immediate acquisition or alternatively fixed in CellFix (BD) for 10 min at 4°C. Absolute cell counts were calculated using CountBright beads (Invitrogen). For apoptosis measurement, cells were washed in Annexin V binding buffer (BD) following surface staining, incubated with Annexin V-APC (BioLegend) for 20 min at 4°C, washed and immediately acquired. For staining of specific γδ TCRs, epidermal cells were rested O/N in complete RPMI at 37°C prior to live/dead dye staining, then were stained with 17D1 hybridoma supernatant 61 at 4°C for 20 min. Cells were then washed and stained with surface antibodies as above including anti-Vγ5-APC (BioLegend) and anti-rat IgM-PE (eBioscience). For analysis of other trypsin-sensitive epitopes, epidermis was separated and digested as above but with TrypLE Express (Gibco) in place of trypsin-GNK, and epidermis was separated for two hours. Cells were stained as above but including combinations of the following antibodies: anti-CD122-PE, anti-4-1BB-APC and anti-4-1BB-PE (all BioLegend). Cells were acquired with an LSR II or Fortessa (BD) and analyzed with FlowJo (Treestar).
Instrument	Data were collected on LSRII or LSR Fortessa instruments (BD)
Software	Data were collected with FACSDiva v8.0.1 (BD) and analyzed with FlowJo v10.3 (Treestar)
Cell population abundance	In preliminary experiments and scRNAseq sorts, purity of sorted cells was >90% as determined by flow cytometry. In subsequent experiments, cells were sorted directly into lysis buffer and so purity could not be assessed.
Gating strategy	Gating strategy figure is shown in Supplementary Figure 1.

☒ Tick this box to confirm that a figure exemplifying the gating strategy is provided in the Supplementary Information.
